# Highly Expressed Genes Are Preferentially Co-Opted for C_4_ Photosynthesis

**DOI:** 10.1093/molbev/msx269

**Published:** 2017-10-11

**Authors:** Jose J Moreno-Villena, Luke T Dunning, Colin P Osborne, Pascal-Antoine Christin

**Affiliations:** Animal and Plant Sciences, University of Sheffield, Sheffield, United Kingdom

**Keywords:** C4 photosynthesis, evolvability, grasses, phylogenetics, transcriptomics, gene co-option

## Abstract

Novel adaptations are generally assembled by co-opting pre-existing genetic components, but the factors dictating the suitability of genes for new functions remain poorly known. In this work, we used comparative transcriptomics to determine the attributes that increased the likelihood of some genes being co-opted for C_4_ photosynthesis, a convergent complex trait that boosts productivity in tropical conditions. We show that independent lineages of grasses repeatedly co-opted the gene lineages that were the most highly expressed in non-C_4_ ancestors to produce their C_4_ pathway. Although ancestral abundance in leaves explains which genes were used for the emergence of a C_4_ pathway, the tissue specificity has surprisingly no effect. Our results suggest that levels of key genes were elevated during the early diversification of grasses and subsequently repeatedly used to trigger a weak C_4_ cycle via relatively few mutations. The abundance of C_4_-suitable transcripts therefore facilitated physiological innovation, but the transition to a strong C_4_ pathway still involved consequent changes in expression levels, leaf specificity, and coding sequences. The direction and amount of changes required for the strong C_4_ pathway depended on the identity of the genes co-opted, so that ancestral gene expression both facilitates adaptive transitions and constrains subsequent evolutionary trajectories.

## Introduction

The evolution of novel physiological adaptations occasionally requires the development of new biochemical cascades, which are generally achieved via the co-option of pre-existing genes into new functions (Duboule and Wilkins 1998; True and Carroll 2002; Monson 2003; Monteiro and Podlaha 2009). Rewiring of biochemical pathways can require both modifications of spatial and temporal gene expression patterns and alterations of the coding sequences (CDSs) to adapt the encoded enzymes to the new catalytic context (Duret and Mouchiroud 2000; Carroll 2008; Aubry et al. 2014). In cases where numerous modifications are needed, the novel pathways can be assembled by natural selection only if a functional version can emerge through relatively few changes, allowing subsequent selection to fix mutations that increase the efficiency of the pathway. Genomic factors that reduce the phenotypic distance between ancestral and novel physiologies, thereby enabling the emergence of novel cascades via few mutations, would consequently be expected to increase accessibility to novel phenotypes. However, in most cases these factors remain poorly understood.

The ability of given genes or genomic features to trigger evolutionary innovation can be investigated via experimental evolution (e.g. Weinreich et al. 2006; Blount et al. 2012), but such studies are restricted to short-lived organisms that do not encapsulate the existing diversity of phyla. For larger organisms with long generation times, a historical approach is the most appropriate. Indeed, phylogenetic inference allows explicit tests of how specific features affect the accessibility of new phenotypes (e.g. Marazzi et al. 2012). Conversely, genomic features that have recurrently contributed to independent origins of a given phenotype can be safely assumed to be suitable for the trait of interest, and their origin can be regarded as potentially facilitating later adaptive transitions (Huang, O’Donnell, et al. 2016). For example, the same autosome pairs were repeatedly co-opted to evolve sex chromosomes in turtles (Montiel et al. 2017), the same gene families encoding crystallins were used to evolve camera eyes in cephalopods and vertebrates (Zinovieva et al. 1999; Yoshida et al. 2014), and homologous genes recurrently contributed to the diversification of coloration patterns in butterflies (Jiggins et al. 2017). Although such evidence indicates that some genomic regions or genes preferentially contribute to specific evolutionary transitions (Tenaillon et al. 2012), multiple factors might increase the adaptive potential, and their identification requires the comparison of the ancestral condition of genes or genomic regions that were recurrently co-opted, to those that were not.

An excellent system to study the factors that increase gene adaptive potential is C_4_ photosynthesis. This novel physiology requires a biochemical cascade arising from the high activity of multiple enzymes in specific leaf compartments, and improves autotrophic carbon assimilation in tropical conditions (Pearcy and Ehleringer, 1984; Hatch 1987; Sage et al. 2012, Atkinson et al. 2016). The C_4_ trait is ecologically and agronomically extremely important (Ehleringer et al., 1997; Still et al., 2003; Byrt et al., 2011). It evolved more than 60 times in independent lineages of flowering plants (Sage et al. 2011), via the co-option of multiple genes that were present in non-C_4_ ancestors (Hibberd and Quick 2002; Aubry et al. 2011; Brown et al. 2011; Kajala et al. 2012). Most enzymes of the C_4_ pathway are encoded by multigene families, whose members differed in their expression patterns and catalytic properties of the encoded enzymes before their involvement in C_4_ photosynthesis (Wang et al. 2009; Hibberd and Covshoff 2010; Aubry et al. 2011; Christin et al. 2013, 2015). Previous comparisons of a handful of C_4_ species have shown that a subset of gene lineages were recurrently co-opted for C_4_ evolution, both among grasses and among the distantly related Caryophyllales (Christin et al. 2013, 2015). However, the co-opted genes differed between grasses and Caryophyllales, suggesting that factors predisposing some genes for a C_4_ function are specific to subgroups of angiosperms (Christin et al. 2015). It has been noted that the co-opted genes appeared to be highly expressed in the non-C_4_ taxa available at the time for comparison, which might have contributed to their preferential co-option (Christin et al. 2013; Emms et al. 2016). However, systematic tests of the factors underlying the observed co-option bias are still lacking.

In this study, we compare transcriptomes across ten independent C_4_ origins in grasses, and their non-C_4_ relatives. Through a combination of phylogeny-based analyses, we test 1) whether a bias in the gene lineages co-opted exists across the whole set of grasses. To determine the causal factors underlying the bias, we then test 2) whether the expression level in leaves and/or 3) whether the tissue specificity in the non-C_4_ ancestors explain variation in the co-option probability among gene lineages. In addition, we analyze CDSs to test 4) whether adaptive changes in the CDSs occurred during or after the emergence of the C_4_ physiology. Together, our investigations shed new light on the factors that increase the adaptive potential of some genes, focusing on a complex trait of ecological and agronomical importance.

## Results

### Sequencing, Read Mapping and Transcriptome Assembly

In total, 74 individually sequenced RNA libraries from 19 species generated over 550 million 100 bp paired-end reads. This represents 98.87 Gb of data, with a mean of 1.34 Gb per library (SD = 0.95 Gb; supplementary table S1, Supplementary Material online). Over 81% of the reads were kept after removing low-quality reads and ribosomal RNA sequences. Transcriptomes were assembled with a mean of 2.23 Gb per species (SD = 1.40 Gb), resulting in a mean of 54,255 Trinity “unigenes” (SD = 17,218.35), 79,566.12 contigs (SD = 23,038.61), and a 1,560.05 bp N50 (SD = 184.95 bp).

The C_4_-related gene families considered in this study constitute 5.1% (SD = 2.02%) of the reads in the leaf libraries of C_4_ plants, versus 2.34% in non-C_4_ plants (SD = 0.75%). On average, 1.05% of the reads from the root libraries mapped to C_4_-related genes (SD = 0.48%).

### Phylogenetic Trees and Identification of Genes Co-Opted for C_4_ Photosynthesis

**Fig. 1.**
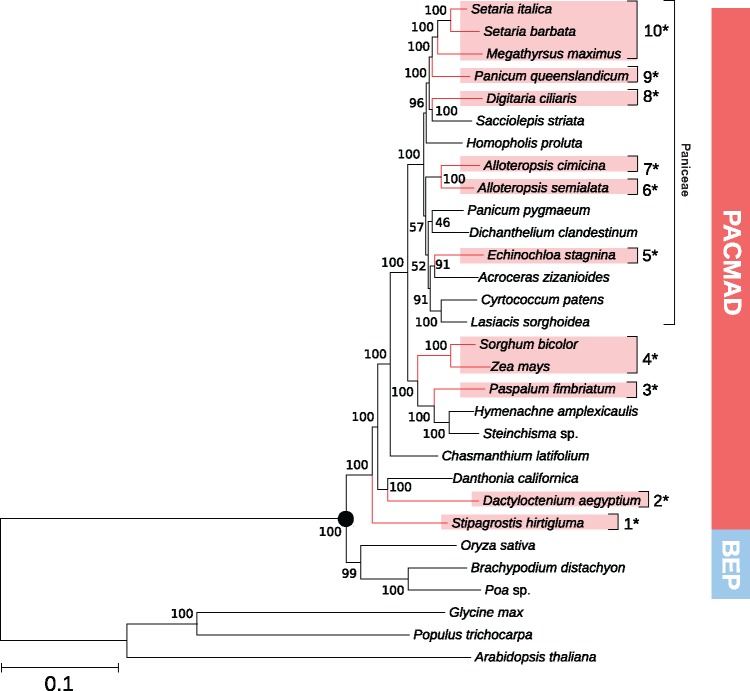
Phylogenetic tree based on nuclear orthologs. C_4_ taxa are in red, and C_4_ origins are numbered. One of the tribe and the two main clades of grasses are indicated on the right. The black circle highlights the node representing the common ancestor of the sampled grasses. Bootstrap values are indicated near branches.

A total of 533 nuclear core-orthologs were used to infer the species tree, which was well resolved (fig. 1). The relationships among grass subfamilies mirror those retrieved previously with other data sets (GPWG II 2012). However, relationships within the Paniceae tribe (the group most densely sampled here) differ in several aspects from those based on plastid markers (GPWG II 2012), and were closer to previous analyses that also included nuclear markers (Vicentini et al. 2008). The placement of the different C_4_ origins within the tree was largely congruent with previous studies, and their non-C_4_ relatives separated them in the phylogeny as expected (fig. 1).

For each gene family encoding C_4_-related enzymes, phylogenetic inference confirmed previous conclusions about orthology (Vilella et al. 2009). The enzyme phosphoenolpyruvate carboxykinase (PCK) and the Na^+^/H^+^ antiporter (NHD) are each encoded by a single gene lineage (supplementary fig. S1, Supplementary Material online). The number of grass co-orthologs in other families varies from two (for pyruvate, phosphate dikinase—PPDK) to eight (for triose phosphate–phosphate translocator—TPT; supplementary fig. S1, Supplementary Material online). Groups of co-orthologs were named as in Christin et al. (2015). Phylogenetic relationships inferred in these gene trees were mostly congruent with the species tree. Exceptions include genes for PCK, where *Echinochloa stagnina* and *Alloteropsis semialata* grouped with those of *Setaria barbata*. This pattern has previously been reported for *Alloteropsis* species and this, together with a number of other lines of evidence, was interpreted as the fingerprint of a lateral gene transfer from *Setaria* or its close relatives (Christin et al. 2012; Dunning et al. 2017). Other incongruences were observed in genes encoding PEPC, PPDK, NAD(P)-malate dehydrogenase [NAD(P)-MDH], Sodium bile acid symporter family (SBAS), TPT, and NDH (supplementary fig S1, Supplementary Material online), and could stem from a combination of reticulate evolution during grass diversification and phylogenetic bias due to adaptive evolution. Gene duplicates specific to subgroups of grasses are evident for several genes, and can in some cases be associated to recent polyploidy (e.g. in *Zea mays* genes *pck-1P1*, *ppc-1P4*, *ppdk-1P2*, *nadmdh-4P7*; supplementary fig. S1, Supplementary Material online). Our analytical pipeline cannot estimate the expression level individually for each of these duplicates with very similar sequences, but these duplications specific to subgroups of grasses are relatively recent and occurred after the divergence of C_3_ and C_4_ clades (supplementary fig. S1, Supplementary Material online). The inferred evolutionary changes in expression patterns and co-option events are consequently not affected.

**Fig. 2.**
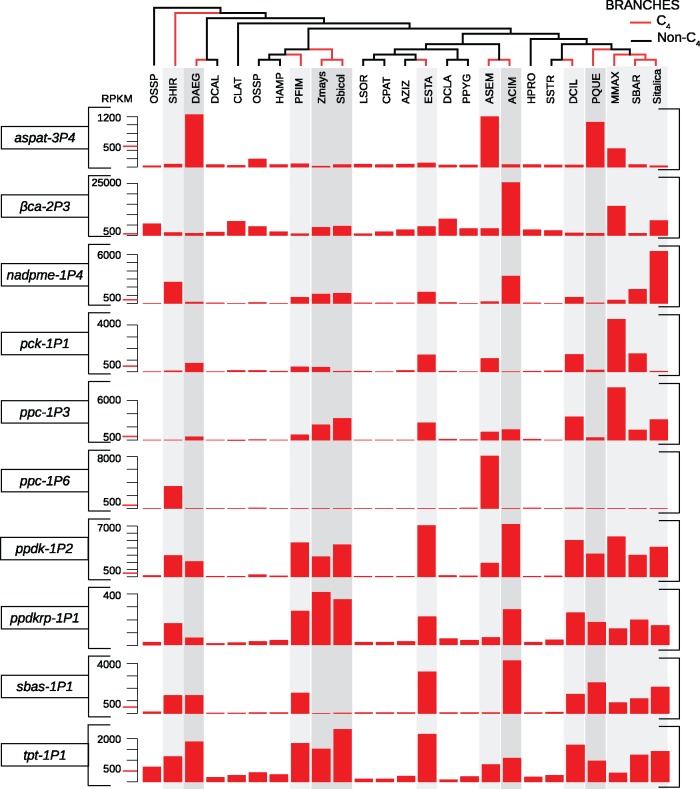
Transcript abundances of the main C_4_ genes in C_4_ and non-C_4_ species. Barplots indicate rpkm values (reads per kilobase per million of reads). Phylogenetic relationships among species are indicated at the top, and C_4_ lineages are numbered as in figure 1, with branches colored in red. Species names are abbreviated as in supplementary tables S1 and S2, Supplementary Material online.

**Fig. 3.**
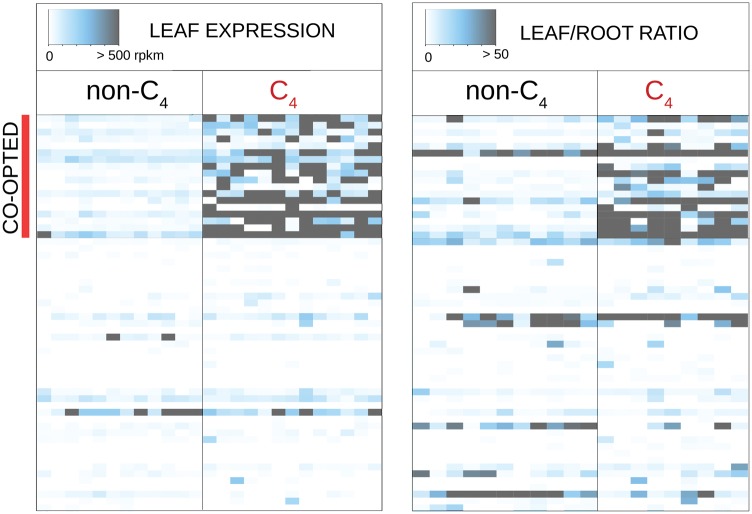
Gene expression profiles of C_4_-related genes in the studied taxa. Colors indicate leaf transcript abundance and leaf/ratio abundance ratio for C_4_-related genes in C_4_ and non-C_4_ species. Genes that have been co-opted at least once are at the top.

The most highly transcribed genes encoding C_4_-related proteins are those for β-carbonic anhydrase (βCA; fig. 2 and supplementary table S2, Supplementary Material online), an enzyme that acts in the cytosol of mesophyll cells in C_4_ plants. These genes are however equally abundant in non-C_4_ species (fig. 2), where the enzyme plays a key role in the chloroplasts of mesophyll cells (Tetu et al. 2007). Of the 31 other gene families encoding enzymes that can be related to the C_4_ pathway, 14 included gene lineages with transcript abundances above 500 rpkm in at least one C_4_ species (fig. 3; supplementary table S2, Supplementary Material online). The transcript abundance of *ppa-4P4* reached 500 rpkm in some C_4_ species, but similar abundance was observed in a number of non-C_4_ taxa (supplementary table S2, Supplementary Material online), and the gene was consequently not counted as C_4_ specific. For the rest of the gene lineages, such high values were not found in non-C_4_ species (supplementary table S2, Supplementary Material online). Genes co-opted for C_4_ photosynthesis were identified in each C_4_ species for most core C_4_ enzymes, but putative C_4_ transporters and regulators were not always abundant in C_4_ leaves (supplementary table S2, Supplementary Material online). Genes for enzymes of the photorespiration pathway were downregulated in C_4_ species, as expected (supplementary table S2, Supplementary Material online).

### Factors Affecting Gene Co-Option

Out of 58 gene lineages encoding the 14 enzymes used by the C_4_ species sampled here, only 18 have been co-opted at least once, and up to ten times independently for *ppdk–1P2* and *tpt–1P1* and eight for *ppc−1P3* (table 1). Given the size of the different gene families and the number of co-option events, fewer genes have been co-opted at least once than expected by chance (*P*-value < 0.00001). This confirms the existence of a co-option bias across the ten C_4_ origins considered here, a result previously reported for Caryophylalles and grasses (Christin et al. 2013, 2015).

**Table 1.** Number of Times a Gene Lineage Was Co-Opted, For Genes Co-Opted at Least Once.

Gene Lineage	Times Co-Opted	Main Catalytic Reaction
*ak-1P1*	8	AMP→ADP
*alaat-1P5*	3	Ala↔Pyruvate
*aspat-2P3*	3	Asp↔OAA
*aspat-3P4*	3	Asp↔OAA
*dit-2P3*	1	Dicarboxylate transporter
*nadpmdh-1P1*	5	Malate↔OAA
*nadpmdh-3P4*	1	Malate↔OAA
*nadpme-1P4*	7	Malate→pyruvate
*nhd-1P1*	5	Sodium proton antiport
*pck-1P1*	5	OAA→PEP
*pepck-1P1*	1	ATP ADP/P antiport
*ppa-1P2.1*	6	Pyrophosphate→phosphate
*ppc-1P3*	8	PEP→OAA
*ppc-1P6*	2	PEP→OAA
*ppdk-1P2*	10	Pyruvate→PEP
*ppt-1P5*	4	PEP phosphate antiport
*sbas-1P1*	8	Pyruvate sodium symport
*tpt-1P1*	10	3-PGA TP antiport

**Fig. 4.**
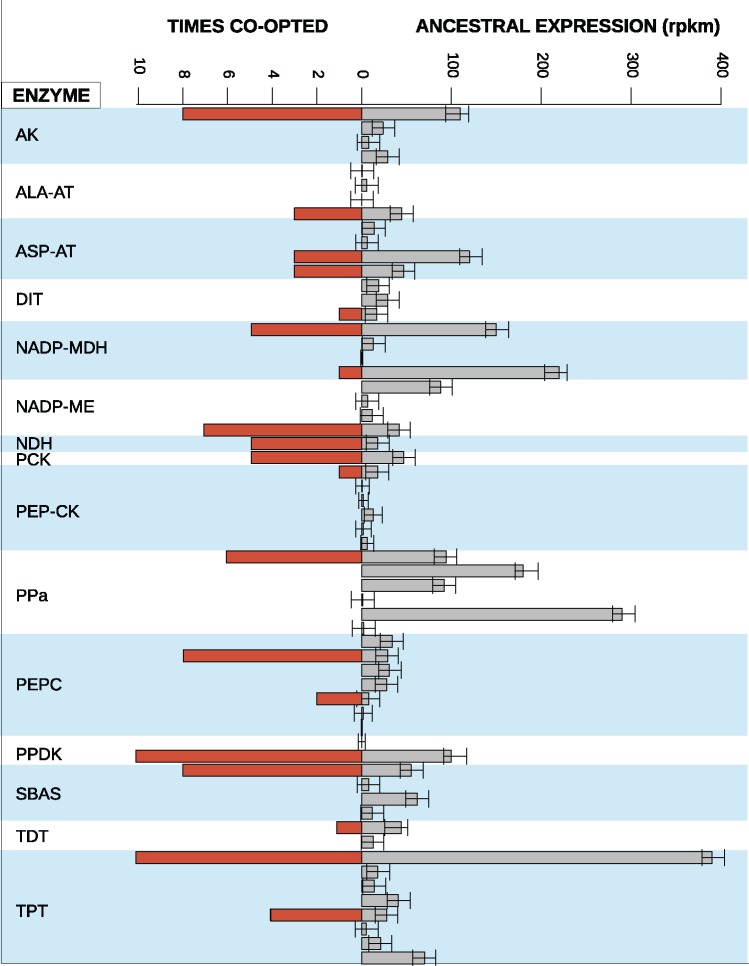
Ancestral leaf transcript abundance and number of co-option events. Barplots on the left indicate the number of times each gene was co-opted, and those on the right indicate the inferred abundance in the non-C_4_ last common ancestor of grasses (see fig. 1), with the associated confidence intervals. Genes are sorted by enyzme, indicated on the left.

The ancestral state reconstructions inferred the abundance in leaves and leaf/root specificity in the last common ancestor of the sampled grasses for each C_4_-related gene (fig. 4). This approach comes with uncertainty, especially for deeper nodes in a tree, but the confidence intervals associated with the inferred values are small compared with the difference among members of the same gene family (fig. 4). The inferred values are moreover tightly correlated with averages of the values among C_3_ grasses (*R*^2^ = 0.98 for the leaf abundance and *R*^2^ = 0.91 for the leaf/root ratio), and were consequently used for modeling of gene co-option. Linear models showed that the ancestral transcript abundance in the leaf significantly affected the co-option frequency (*F* = 13.11, df = 56, *P* = 0.0006336; *R*^2^ = 0.19), and this stayed significant when the gene family was used as a co-factor (table 2). The effect of the ancestral leaf/root transcript abundance ratio on the co-option frequency was not significant when considered on its own (*F* = 0.40, df = 56, *P* = 0.54), or in combination with the ancestral leaf abundance and the gene family cofactor (table 2). Therefore, our modeling analyses indicate that genes were co-opted for C_4_ photosynthesis based on their transcription level in leaves (fig. 4), independently of the specificity of this expression in leaves compared with roots. The same conclusions were reached when using a threshold of 300, 1,000, and 1,500 rpkm for the identification of co-opted genes (see table 2).

**Table 2.** Results of Analyses of Variance on Linear Models of Number of Co-Option Events Based on Ancestral Leaf Abundance (ala), Leaf/Root Ratio, and Gene Family Identity (family), with Co-Opted Genes Identified with Different rpkm Thresholds.

rpkm Threshold	300	300	300	500	500	500	1,000	1,000	1,000	1,500	1,500	1,500
Factors	ala	Leaf/Root	Family	ala	Leaf/Root	Family	ala	Leaf/Root	Family	ala	Leaf/Root	Family
***P*-value**	0.00	0.52	0.38	0.00	0.57	0.56	0.00	0.88	0.21	0.01	0.77	0.10
**df**	1, 42	1, 42	13, 42	1, 42	1, 42	13, 42	1, 42	1, 42	13, 42	1, 42	1, 42	13, 42
***F*-statistics**	17.07	0.78	0.95	12.65	0.32	0.90	14.46	0.21	1.37	8.29	0.0.09	1.71

Note.—df, degrees of freedom. For each variable, the degrees of freedom for the residuals are given after the comma.

Transcriptome data sets for clades containing C_3_ and C_4_ species other than grasses are focused on small taxonomic groups, so that ancient evolutionary events cannot be inferred yet outside from grasses. A test using published transcriptomes for one C_3_ and C_4_ species within the eudicot family Cleomaceae failed to detect any effect of expression levels on the identity of genes co-opted for C_4_ (supplementary tables S3 and S4, Supplementary Material online), but the availability of a single C_4_ origin and only one C_3_ relative likely decreased statistical power. Although the same statistical limitations applied to the *Flaveria* data set, our preliminary investigation suggested that the effect of leaf abundance on the co-option probability might apply to multiple C_4_ origins across the angiosperms. Indeed, there was a significant effect of the leaf abundance in the close relatives on the co-option probability for *Flaveria* (supplementary table S4, Supplementary Material online).

### Marked Differences in Transcript Abundance and CDSs

Although the ancestral transcript abundance significantly affects the probability of a gene being co-opted, the evolution of C_4_ photosynthesis is accompanied by major increases in transcript abundance. The transcripts of genes encoding C_4_ enzymes increase by a fold change of up to 480 for *ppc–1P6* in *A. semialata* compared with related non-C_4_ taxa (fig. 2). In addition, their leaf specificity increases, to reach leaf/root ratios of up to 6,204 after their co-option into C_4_ photosynthesis, compared with a maximum of 257 in non-C_4_ taxa (fig. 3).

**Fig. 5.**
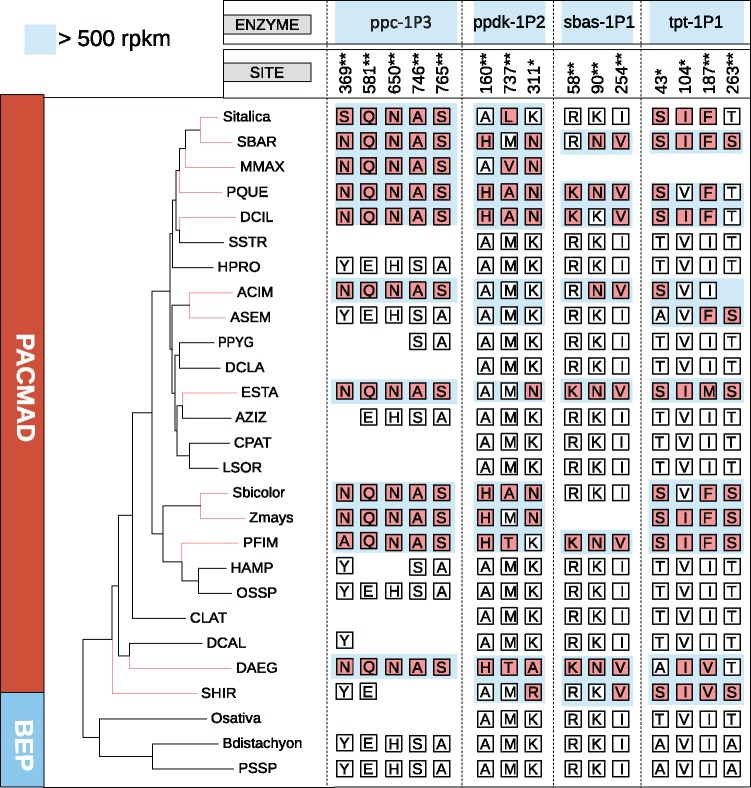
Patterns of convergent adaptive amino acid replacements. The phylogeny of the sampled species is indicated on the left, with species names abbreviated as in supplementary table S1, Supplementary Material online. Branches leading to C_4_ species in red. Amino acids at some of the sites under positive selection (*P < 0.05; **P < 0.01) are indicated on the right. Residues of co-opted genes are highlighted with a blue background.

Besides these changes in transcript abundance, tests for positive selection revealed adaptive evolution in the CDSs of a number of genes during or slightly after their co-option into C_4_ photosynthesis. After correction for multiple testing, the test for a shift of selective pressures along C_4_ branches (A1 vs. M1a comparison) was significant for nine genes out of 19 (supplementary table S5, Supplementary Material online). The test specifically testing for a shift to positive selection as opposed to a relaxation of selection (A1 vs. A comparison) was also significant for four of these nine genes; *ppc-1P3*, *ppdk-1P2*, *sbas-1P1*, *and tpt-1P1* (supplementary table S5, Supplementary Material online). The sites identified by the Bayes Empirical Bayes analysis as being under positive selection along C_4_ branches showed widespread cases of parallel amino acid replacements (fig. 5).

## Discussion

### Expression Patterns Determined Which Genes Were Co-Opted for C_4_

In this study, we analyzed root and leaf transcriptomes from grass species representing ten independent origins of C_4_ photosynthesis as well as the close non-C_4_ relatives to each of them (fig. 1). As previously suggested based on smaller species samples (Christin et al. 2013, 2015), the co-option of genes for the C_4_ pathway has been a nonrandom process. Indeed, despite multiple gene lineages existing for most C_4_-related enzymes, a few of them were co-opted more frequently than expected by chance, while most were never used in the ten C_4_ lineages evaluated here (table 1 and figs. 3 and 4). A number of factors could explain the preferential co-option of some genes for a novel function, including their availability via genomic redundancy, the suitability of their kinetic properties, the fit of their expression patterns, and their evolvability (Aharoni et al. 2005; Landry et al. 2007; Christin et al. 2010, 2015; Stiffler et al. 2015; Huang, O’Donnell, et al. 2016). Our approach was specifically designed to test for the effects on co-option probability of two dimensions of the expression patterns inferred for non-C_4_ ancestors; the transcript abundance in leaves and the leaf versus root specificity. Thanks to the evolutionary-informed sampling (fig. 1), we were able to unambiguously show that the likelihood of gene co-option into C_4_ photosynthesis was determined in a large part by their transcript abundance in leaves prior to C_4_ evolution (fig. 4), with no apparent effect of the leaf to root specificity (table 2).

The C_4_ biochemical pathway, like any complex pathway, is assumed to result from many rounds of fixation of adaptive mutations (Sage et al. 2012; Heckmann et al. 2013; Dunning et al. 2017). However, natural selection cannot gradually improve a pathway before it exists, even in a rudimentary stage (Huang, O’Donnell, et al. 2016). It is likely that a primitive, weak C_4_ cycle initially emerged in some species via a slight upregulation of few genes, as observed in intermediate plants accumulating only part of their CO_2_ via the C_4_ cycle (Mallmann et al. 2014; Dunning et al. 2017). We show here that some genes were already moderately abundant in leaves of non-C_4_ plants (fig. 4), a pattern that likely evolved for a number of reasons not related to C_4_ photosynthesis, but eased its later evolution. This facilitator effect would have been even stronger if C_4_-related genes were upregulated in the low-CO_2_ conditions that prevailed until the Industrial Revolution, as has been suggested for the distantly related *Arabidopsis* (Li et al. 2014). The encoded enzymes, present in the leaves of the non-C_4_ ancestors, constituted the building blocks needed to generate a weak, yet functional, C_4_ pathway following key mutations. These could have included further upregulation of key C_4_ enzymes or alterations of the leaf structural arrangements, pushing the system beyond a tipping point where the C_4_ pathway could emerge. Models predict that, once a C_4_ pathway is in place, any increase in the rate of the C_4_ pathway will increase productivity in warm conditions (Heckmann et al. 2013; Mallmann et al. 2014). Any rudimentary C_4_ pathway based on ancestrally abundant enzymes would therefore have created the selective impetus for upregulation of enzymes, generating the striking patterns observed in derived C_4_ plants (figs. 2 and 3).

Besides elevated abundance of numerous enzymes, the C_4_ trait is characterized by a precise compartmentalization of the biochemical reactions in different parts of the leaves (Hatch and Osmond 1976; Hatch 1987; John et al. 2014). Interestingly, transcript abundance in nonphotosynthetic tissues, such as roots, did however not prevent the co-option of a gene lineage for C_4_ photosynthesis (table 2 and fig. 3), and previous pairwise comparisons have established that orthologs to C_4_ genes have a diversity of expression patterns in non-C_4_ species (Külahoglu et al. 2014). We conclude that being abundant in leaves was a sufficient condition for the C_4_ function, independently of the presence in other tissues. Cellular and subcellular localization, which was not captured by our whole-leaf transcriptomes, probably still contributed to determining which genes were co-opted for C_4_. For instance, only one of the four gene lineages for NADP-ME present in grasses encodes a chloroplast-specific isoform, and this gene lineage has been recurrently co-opted for C_4_ despite an ancestral abundance of a second gene (fig. 4; Christin et al. 2009). Similarly, the product of *ppc-1P2*, the most highly expressed gene for PEPC in non-C4 plants (fig. 4), is chloroplast-specific (Masumoto et al. 2010), which very likely prevented a function in C_4_ photosynthesis, since this enzyme is cytosolic in the C_4_ pathway. Independently of these specific cases, the mere moderate abundance in leaves explains a large fraction of the co-option probability.

### Despite Genetic Enablers, C_4_ Evolution Required Massive Changes

Our study is the first to scan the transcriptomes of a number of non-C_4_ grasses closely related to C_4_ species, and showed that genes co-opted for C_4_ tended to already be abundant in non-C_4_ ancestors (figs. 3 and4). Although transcriptomes in other groups are not available for multiple C_4_ origins and their C_3_ relatives, our reanalysis of eudicot data sets suggested that the preferential co-option of the most abundant genes might underly C_4_ origins in groups other than grasses (supplementary table S4, Supplementary Material online). This suggests that the abundance of some enzymes able to fulfil a C_4_ function facilitated the emergence of a C_4_ pathway. However, massive changes in gene expression are still observed between non-C_4_ and C_4_ relatives (e.g. Bräutigam et al. 2011, 2014; Külahoglu et al. 2014). Indeed, genes encoding C_4_ enzymes are orders of magnitude more abundant in C_4_ leaves, and leaf specificity strongly increased after the co-option of genes for C_4_ (figs. 2 and3). In addition, evidence for widespread adaptive evolution of CDSs for the C_4_ context, obtained here and in other studies (fig. 5; Besnard et al. 2009; Christin et al. 2009; Wang et al. 2009; Huang, Studer, et al. 2016), suggests important modifications of the kinetic properties, shown for some enzymes (Bläsing et al. 2000; Tausta et al. 2002). Instead of being involved in the initial emergence of a C_4_ cycle, we propose that these massive changes were involved in the transition from a weak to a strong C_4_ pathway able to match the high rates of the Calvin cycle, as suggested for specific study systems (Svensson et al. 2003; Mallmann et al. 2014; Dunning et al. 2017).

Since the major requirement for a C_4_ function was sufficient abundance in leaves, the co-opted genes were not necessarily the best suited for the C_4_ function, in terms of the tissue specificity or kinetic properties of the encoded enzyme. The ancestral abundance might therefore have constrained the initial emergence of a weak C_4_ cycle based on specific sets of genes, forcing natural selection to later adapt their properties to those required for a high-flux strong C_4_ cycle. The recurrent co-option of the same co-orthologs would have increased the likelihood of adaptation via similar changes, explaining the observed parallel amino acid replacements among C_4_ origins in grasses (fig. 5; Christin et al. 2007). It has been shown that C_4_ lineages belonging to distant groups of angiosperms in some cases co-opted distinct genes (Christin et al. 2015; supplementary table S4, Supplementary Material online). Because of the large evolutionary distances separating these groups, which are further increased when different co-orthologs are co-opted (supplementary table S4, Supplementary Material online), the encoded enzymes likely varied in their kinetic properties in addition to their leaf and cell specificities. The amount of optimizing adaptive changes might have varied among major C_4_ groups as a consequence, explaining that the frequency and identity of selection-driven amino acid replacements shows high convergence among closely related C_4_ lineages (fig. 5), but varies between C_4_ origins in grasses and those in the distantly related sedges and eudicots (Besnard et al. 2009).

## Conclusions

In this study, we sequenced the transcriptomes of species from the main C_4_ grass lineages as well as their close non-C_4_ relatives, and used models to show that the identity of genes co-opted for C_4_ photosynthesis was largely explained by transcript abundance before C_4_ evolution. The co-option, likely dictated by the mere presence of each protein in leaves, was followed by massive upregulation and widespread adaptation of CDSs. Both of these processes likely accelerated and optimized a C_4_ pathway that initially emerged from the combined action of enzymes already present in leaves. It is currently unknown why some gene lineages came to be more expressed than others in non-C_4_ plants but, despite variation among species, the increased abundance of these genes seems to date back to at least the last common ancestor of grasses. Comparison among distant groups of angiosperms indicates that the preferential co-option of the most abundant gene lineages might be a recurrent pattern, but the sampling is not yet dense enough across angiosperms to precisely determine when increased transcript abundance first happened, among the ancestors of grasses and other groups that recurrently evolved C_4_ photosynthesis. When this information is available, we might be able to test whether gene abundance combined with anatomical variation determined which plant lineages were more likely to evolve C_4_ photosynthesis, once environmental changes created the selective pressure for this physiological novelty.

## Materials and Methods

### Species Sampling

Grass species were selected for analyses based on their photosynthetic type to include multiple C_4_ origins and their non-C_4_ relatives, based on previous phylogenetic analyses (GPWG II 2012). We sequenced eight C_4_ species and eleven non-C_4_ species, which separate them in the phylogenetic tree of grasses (GPWG II 2012, fig. 1). Most of these belong to the PACMAD clade (subfamilies Panicoideae, Arundinoideae, Chloridoideae, Micrairoideae, Aristidoideae, and Danthonioideae), which contains all C_4_ origins in grasses, and one non-C_4_ Pooideae species was added as an outgroup for comparisons.

The selected species were grown from seeds, using the material from Atkinson et al. (2016) and Lundgren et al. (2015). Plants were maintained in controlled environment growth chambers (Conviron BDR16; Manitoba, Canada), with 60% relative humidity, 500 µmol m^−2^ s^−1^ photosynthetic photon flux density, and 25/20 °C day/night temperatures, with a 14-h photoperiod. John Innes No. 2 potting compost (John Innes Manufacturers Association, Reading, England) was used. Plants were watered three times a week to keep the soil damp, and were fertilized every 2 weeks with Scotts Evergreen Lawn Food (The Scotts Company, Surrey, England). After a minimum of 30 days in these controlled conditions, two young roots and the most photosynthetically active distal half of fully expanded leaves were sampled from two individuals of each species (biological replicates) during the middle of the photoperiod, and immediately frozen in liquid nitrogen. All samples were stored at −80 °C until RNA extraction.

### RNA Extraction, Sequencing, and Transcriptome Assembly

Samples were homogenized in liquid nitrogen using a pestle and a mortar, and RNA was extracted using the RNeasy Plant Mini Kit (Qiagen, Hilden, Germany), following the manufacturer’s instructions. The isolated RNA was DNA digested on-column using the RNase-Free Dnase Set (Qiagen, Hilden, Germany) and eluted in RNAse-free water with 20 U/µl of SUPERase-IN RNase Inhibitor (Life Technologies, Carlsbad, CA). Extractions that yielded an RNA integrity number (RIN) >6.5 and at least 0.5 µg of total RNA, as determined with the RNA 6000 Nano kit with an Agilent Bioanalyzer 2100 (Agilent Technologies, Palo Alto, CA), were used for upstream procedures. Individual RNA libraries were prepared using TruSeq RNA Library Preparation Kit v2 (Illumina, San Diego, CA), following the manufacturer’s protocol with a target median insert length of 155 bp. A total of 24-indexed libraries were pooled per lane of flow cell and sequenced on an Illumina HiSeq 2500 platform with 100 cycles in rapid mode generating 100 bp paired-end reads, at the Sheffield Diagnostic Genetics Service.

Reads were filtered and assembled using the Agalma pipeline version 0.5.0, with default parameters (Dunn et al. 2013). This pipeline removes low quality reads (Q < 33), and those that are adaptor-contaminated or correspond to ribosomal RNA. The filtered reads are then used for de novo assembly using Trinity (version trinityrnaseq_r20140413p1; Grabherr et al. 2011). One assembly was generated per species, using all the libraries available. Leaf assembly and reads in duplicates from the C_4_*Alloteropsis cimicina* were retrieved from Dunning et al. (2017), and reads for the C_4_*Megathyrsus maximus* and the non-C_4_*Dichanthelium clandestinum*, in triplicates and without replicate, respectively, were retrieved from Bräutigam et al. (2014). RNA-seq reads for C_4_ grasses with a completely sequenced genome were also retrieved from the literature (*Setaria italica* without replicate from Zhang et al. [2012], *Z. mays* without replicate from Liu et al. [2015], and *Sorghum bicolor* in duplicates from Fracasso et al. [2016]). The final RNA expression data set included 12 non-C_4_ species and 13 C_4_ species of grasses.

### Inference of a Species Tree Based on Core Orthologs

CDSs were predicted from the assembled contigs and those retrieved from the literature using the standalone version of OrfPredictor (Min et al. 2005). Protein sequences of eight publicly available genomes (*Arabidopsis thaliana*, *Brachypodium distachyon*, *Glycine max*, *Oryza sativa*, *Populus trichocarpa*, *S. italica, So. bicolor*, and *Z. mays*) were used as references to improve the identification of open reading frames by providing the program with a precomputed BLASTX output file, using parameters suggested by the authors (Min et al. 2005). CDS from contigs with “no hit” in the BLASTX output were predicted ab initio. The predicted CDS were used for subsequent analyses.

CDS homologous to an a priori defined set of plant genes were retrieved using a Hidden Markov Model based search tool (HaMSTR v.13.2.3; Ebersberger et al. 2009). The set of genes includes 581 single copy core-orthologs from plants and is derived from the Inparanoid ortholog database (Sonnhammer and Östlund 2014), using five high quality genomes (*A. thaliana*, *Vitis vinifera*, *O. sativa*, *So. bicolor*, and *Ostreococcus lucimarinus*). Sequences were aligned as described in Dunning et al. (2017); alignments shorter than 100 bp after trimming were discarded, and alignments including sequences from at least ten species were concatenated. The resulting alignment was used to infer a maximum likelihood tree with Phyml (Guindon and Gascuel 2003), using a GTR + G + I nucleotide substitution model, which was identified as the best model using the Smart Model Selection (Lefort et al. 2017). Support was evaluated by 100 bootstrap pseudoreplicates.

### Identification of Homologs and Grass Co-Orthologs Encoding C_4_-Related Enzymes

For each gene family that encodes enzymes related to the C_4_ pathway (identified based on the literature; Mallmann et al. 2014; Li et al. 2015), homologous CDS were retrieved from three publicly available genomes (*S. italica, So. bicolor*, and *A. thaliana*), based on the annotation and previously inferred homology (Vilella et al. 2009). The same approach was used to analyze genes of the photorespiration pathway, which are expected to be downregulated during C_4_ evolution (Mallmann et al 2014). CDS from the sequenced transcriptomes or retrieved from the literature that were homologous to any sequence in each gene family were identified via BLAST searches. Positive matches with a minimal e-value of 0.01 and minimal mapping length of 500 bp were retrieved and added to the data sets. Only the first transcript model was considered for complete genomes, and the longest CDS from each set of Trinity gene isoforms was used.

A new alignment was produced for each gene family ensuring high-quality alignments while maintaining as many sites as possible. This approach requires manual curation, and was consequently not used for the 581 sets of core orthologs described earlier. A preliminary alignment was obtained for each gene family using MUSCLE (Edgar 2004). The alignment was manually inspected in MEGA version 6 (Tamura et al. 2013), and potential chimeras and sequences of ambiguous homology (false positives) identified through visual inspection and comparison with other sequences were removed. The remaining sequences were re-aligned as codons using ClustalW (Thompson et al. 1994), and the alignments were manually refined. For each gene family, the alignment was used to compute a maximum likelihood phylogenetic tree, using PhyML (Guindon and Gascuel 2003), and the GTR + G + I substitution model as best-fit model identified previously for most of the gene families in this study (Christin et al. 2015). Support values were evaluated with 100 bootstrap pseudoreplicates.

Groups of grass co-orthologs, which include all the genes that descend from a single gene in the last common ancestor of grasses through speciation and gene or genome duplications (including the ancient polyploidy in the common ancestor of grasses; Tang et al. 2010), were identified based on the phylogenetic trees inferred for each gene family. Duplicates specific to some groups of grasses, which might have emerged via gene or genome duplication (whether via auto- or allopolyploidy) after the diversification of grasses, would be grouped in the same co-orthologs, so that our orthology assessment and subsequent expression analyses are not influenced by polyploidization events. Cleaned reads were mapped back to sequences belonging to any of the gene families as single reads, using the local alignment option in Bowtie2 (Langmead and Salzberg 2012). Our approach allows reads to map back to sequences from the same species, but also allows sequences from other closely related species to serve as the reference. The number of reads mapped to each group of co-orthologs was reported as reads per kilobase of aligned exons per million of cleaned reads (rpkm). These proxies for transcript abundances were obtained for each replicate.

### Identification of Co-Opted Genes and Factors Increasing Co-Option Rates

Enzymes of the C_4_ pathway are abundant in the leaves of C_4_ species because high catalytic rates are needed to match the fluxes of the Calvin cycle (Furbank et al. 1997, Mallmann et al. 2014). Transcripts encoding enzymes known to act in the C_4_ pathway were consequently identified as those that reached an abundance of at least 500 rpkm in leaves of a given C_4_ species. Because this threshold is arbitrary, subsequent analyses were repeated with other thresholds (300, 1,000, and 1,500 rpkm), which did not affect our conclusions (see “Results” section). Previous investigations comparing a limited number of species have shown that, within a given taxonomic group, independent C_4_ origins tend to co-opt the same gene lineages (Christin et al. 2013, 2015; Emms et al. 2016). To test this expectation across our larger species sample, the number of genes co-opted at least once in our data set was compared with the number expected by chance given the size of the different gene lineages and the number of co-option events, following the resampling approach of Christin et al. (2015).

Once a bias in gene co-option was confirmed (see Results), we tested for factors potentially affecting the probability of a given group of co-orthologs being co-opted for C_4_. We used the values inferred for the last common ancestor of grasses as proxies for the condition before C_4_ evolved, with two different dimensions of the expression patterns. First, we inferred the leaf transcript abundance. Second, we inferred the leaf/root ratio of abundances as a proxy for leaf specificity. For each group of co-orthologs, the values of these variables in the common ancestor of grasses were estimated using the phylogeny obtained with HaMSTR and the “ace” function in the R package “ape” version 3.5 (Paradis et al. 2004). The maximum likelihood method was selected, with a Brownian motion model. In this approach, the value of the continuous variable that maximizes the likelihood is calculated for each node, with the associated confidence intervals. Only non-C_4_ species were included in the ancestral state analyses to avoid biases caused by high levels in C_4_ taxa. Considering only the gene families co-opted at least once, linear models, as implemented in the “lm” function in R version 3.3.2 (R Development Core Team 2016), were used to test independently for an effect of ancestral leaf transcription abundance and of ancestral leaf/root ratio on the number of times each group of co-orthologs has been co-opted. An analysis of variance on multiple linear models was then used to determine whether the effect of ancestral leaf abundance and/or leaf/root ratio remain when the gene family is included as a co-factor.

Transcriptome data sets available for groups of closely related C_3_ and C_4_ species outside of grasses were used to assess whether the observed patterns are valid across flowering plants. Data for one C_3_ and one C_4_ Cleomaceae were retrieved from Bräutigam et al. (2011), and the phylogenetic annotation of C_4_-related genes in these data sets was deduced from the identity of orthologs from the closely related *Arabidopsis* and the phylogenetic trees from Christin et al. (2015). For *Flaveria*, RNAseq data were retrieved for two C_3_ species from Mallmann et al. (2014) and for one C_4_ species from Lyu et al. (2015). The reads were annotated in the original study based on their similarity to *Arabidopsis* sequences, but the evolutionary distance between *Flaveria* and *Arabidopsis* can potentially mislead orthology assessments. We consequently performed de novo assemblies using the published reads, and obtained the transcript abundance for C_4_-related genes using the previously published phylogenetic annotation pipeline (Christin et al. 2015). Groups of co-orthologs co-opted for C_4_ by *Flaveria* or Cleomaceae were identified based on the literature (reviewed in Christin et al. 2015) or based on leaf abundance reaching 500 rpkm in C_4_ species for the genes not included in previous reviews. The effect of the abundance in the C_3_ relatives on the co-option probability was modeled as for grasses, independently for Cleomaceae and *Flaveria*. Because two C_3_ species are available for *Flaveria*, their average abundance was used. Root abundance was not available for the same species, so that the effect of leaf specificity in these groups of eudicots could not be tested.

### Positive Selection Tests

Codon models were used to test for positive selection following the co-option of genes for C_4_ photosynthesis. For each group of co-orthologs that has been co-opted at least once for C_4_, the inferred alignment was truncated as needed to remove poorly aligning ends and a new phylogenetic tree was inferred with phyML, considering only third positions of codons to remove potential biases due to adaptive evolution. The inferred topology was used to optimize three different codon models, using codeml as implemented in PAML (Yang 2007). These models rely on the ratio of nonsynonymous mutation rate per synonymous mutation rate (ω; Yang and Nielsen 2002, 2008; Yang and Swanson 2002). In the null model M1a, codons evolve under either purifying or relaxed selection in all branches (ω smaller than and equal to one, respectively). In the branch-site models, some codons still evolve under neutral or purifying selection in all branches, but others shift from purifying or relaxed selection in background branches to relaxed (in model A) or positive (in model A1) selection in foreground branches. These foreground branches are defined a priori. In our case, all branches descending from each C_4_ co-opted gene (identified above for the species sequenced here and from the literature for the rest of species) were set as the foreground branches. Because genes for βCA were present at similar abundance in non-C_4_ and C_4_ species (see “Results” section), but these are known to be part of the C_4_ pathway (Budde et al.1985; Hatch and Burnell 1990), all branches leading to C_4_ species in these gene families were selected as foreground branches. The fit improvement of the model assuming changes in selection pressures was evaluated using likelihood ratio tests. The model A1 was first compared with the model M1a, to test for selective shifts following the co-option event, and then to the model A to specifically test whether the shift corresponded to positive selection. *P*-values were corrected for multiple testing.

## Supplementary Material

Supplementary data are available at *Molecular Biology and Evolution* online.

## Supplementary Material

Supplementary Data
