# Supplementary material for: Highly Expressed Genes Are Preferentially Co-Opted for C4 Photosynthesis
Source: Mol Biol Evol. 2017 Oct 11;35(1):94–106. doi: 10.1093/molbev/msx269 (PMC5850498; doi:10.1093/molbev/msx269)
Supplement: Supplementary Data [file msx269_supp.zip › msx269_supp.pdf]

Supplementary Material for:

"Highly expressed genes are preferentially co-opted for C_4 photosynthesis"

by Jose J. Moreno-Villena, Luke T. Dunning, Colin P. Osborne, Pascal-Antoine Christin

This Supplementary Material contains one figure and four tables:

Figure S1: Phylogenetic trees for C_4-related gene families.

Table S1: Sequencing statistics.

Table S2: Transcript abundance of C_4-related genes in the different samples.

Table S3: Gene expression levels and co-option in two groups of eudicots

Table S4. Statistical models of co-option events for two groups of eudicots.

Table S5. Results of codon models comparisons.

Figure S1: Phylogenetic trees for C_4-related gene families.

For each gene family with members potentially involved in the C_4 or photorespiratory pathways, a maximum phylogenetic tree is shown. Name of the enzyme is indicated at the top. Bootstrap support values are indicated near nodes, when higher than 50. Gene lineages are indicated on the right, with brackets.

Aspartate aminotransferase (ASP-AT)

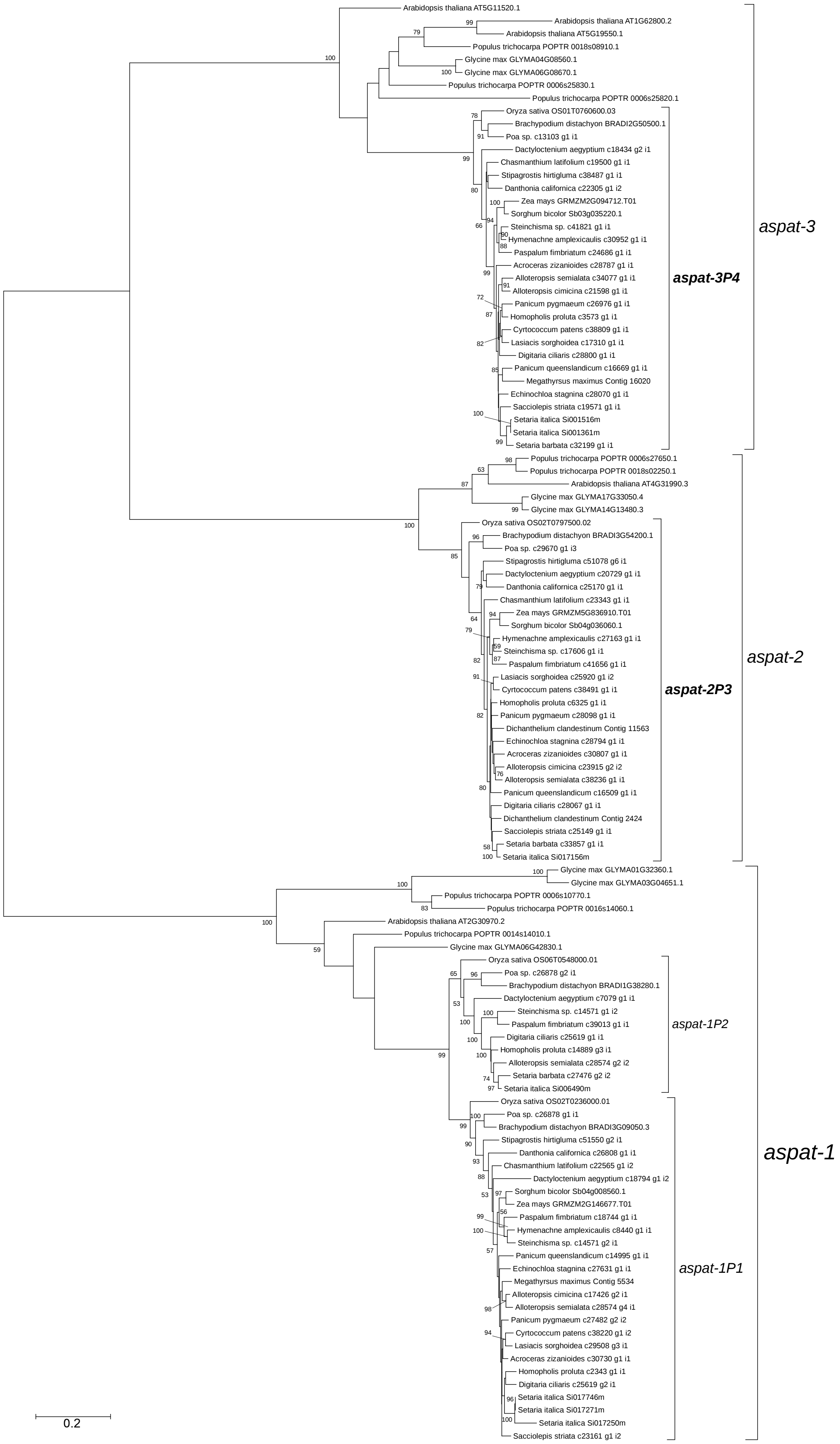

Phosphoenolpyravute carboxykinase (PCK)

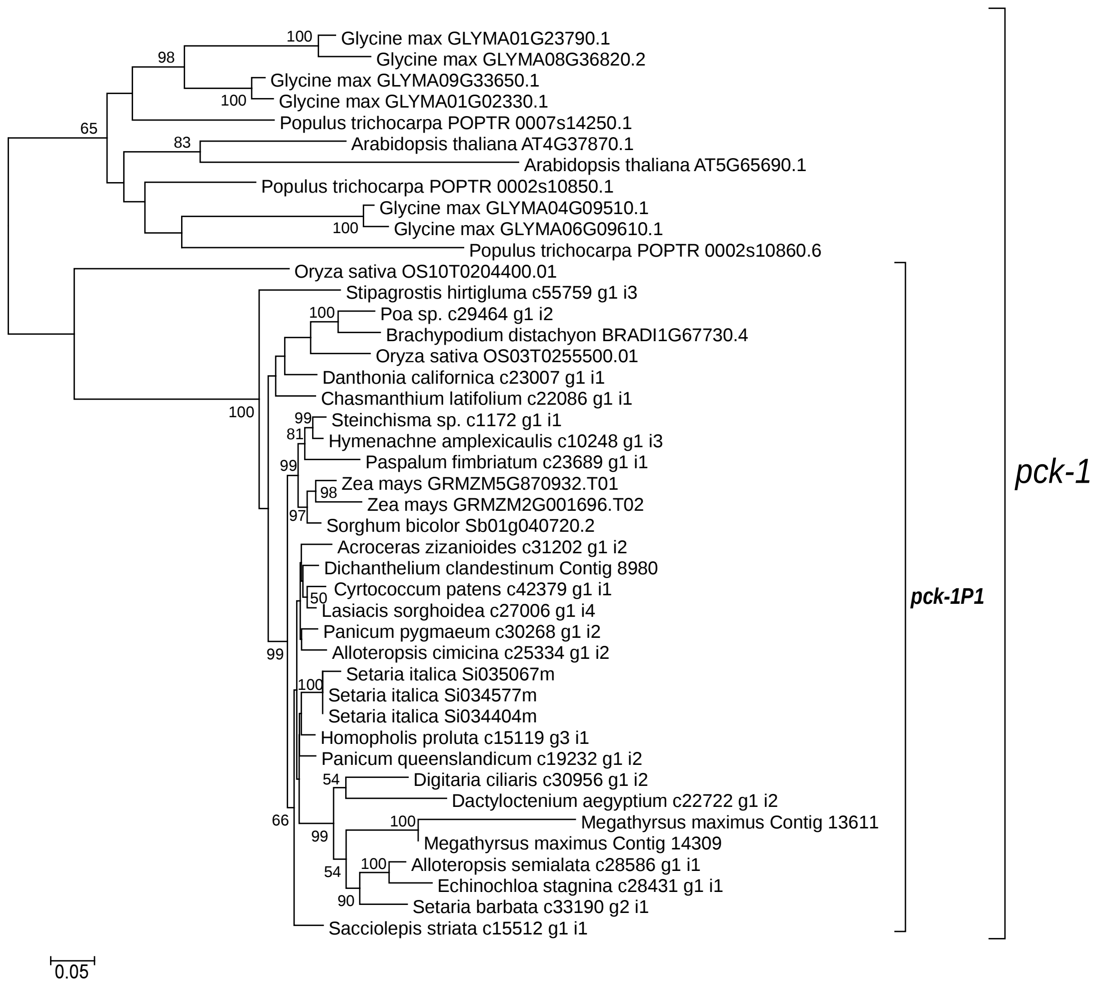

Phosphoenolpyruvate carboxylase (PEPC)

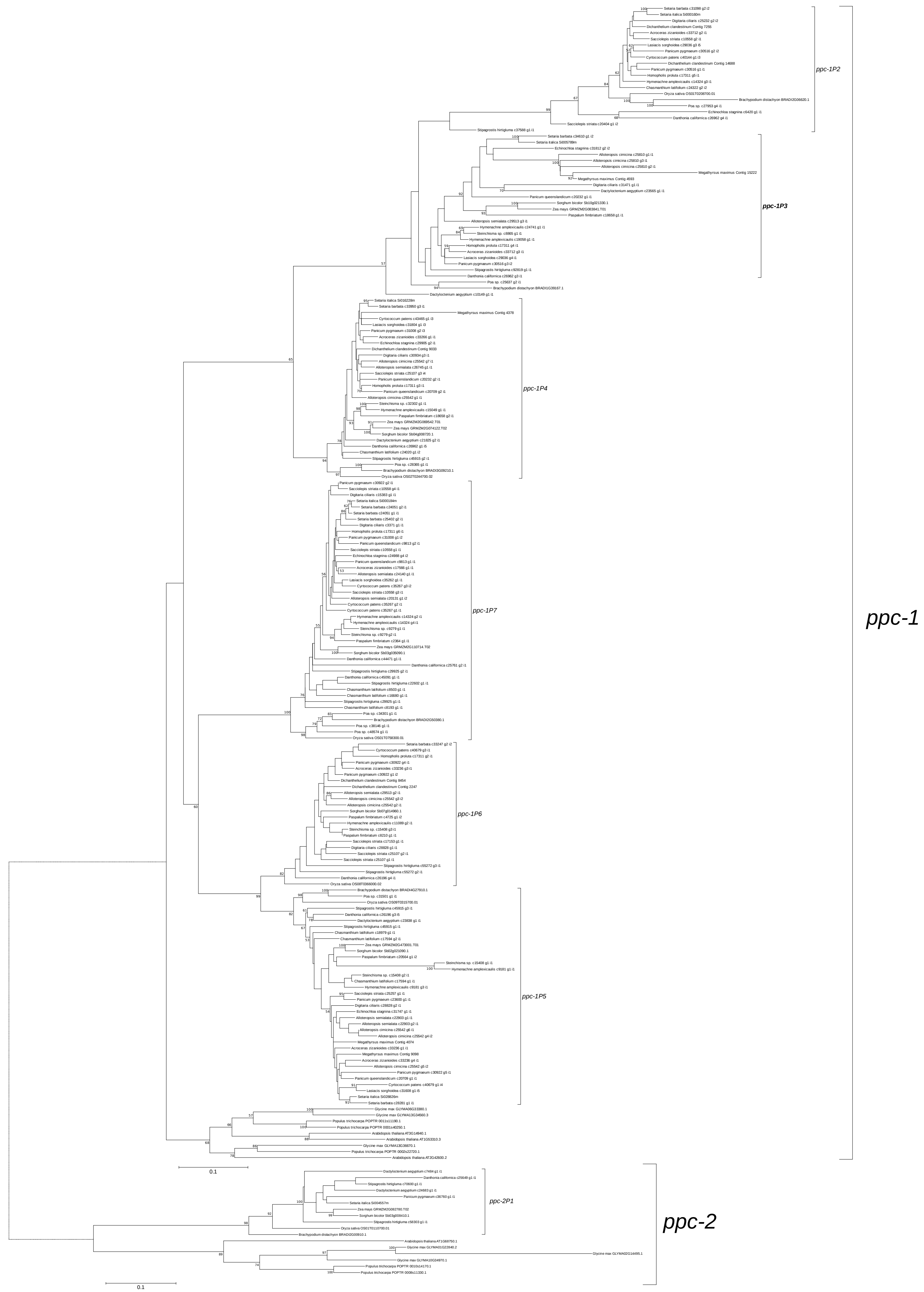

Pyruvate, phosphate dikinase (PPDK)

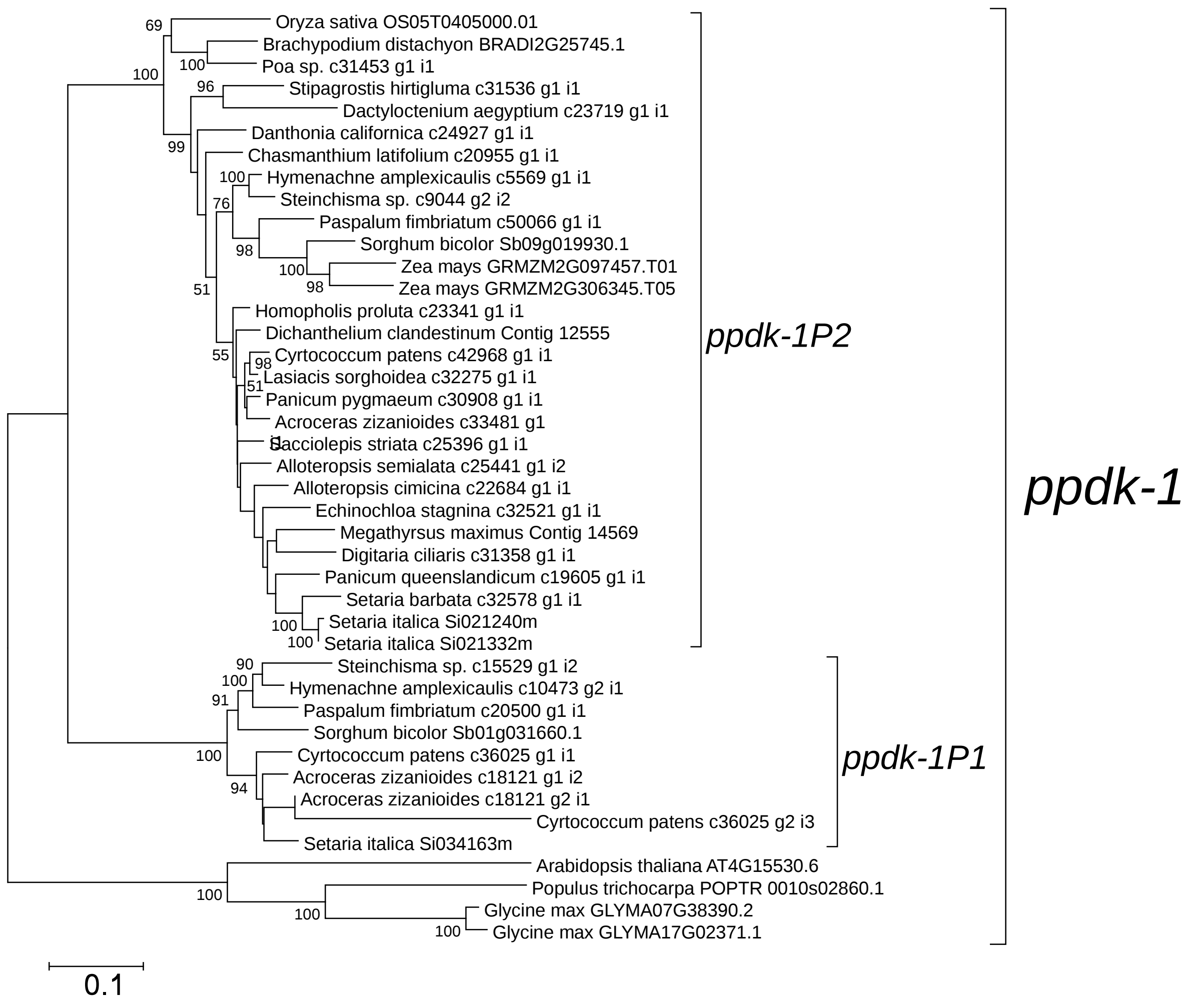

Alanine aminotransferase (ALA-AT)

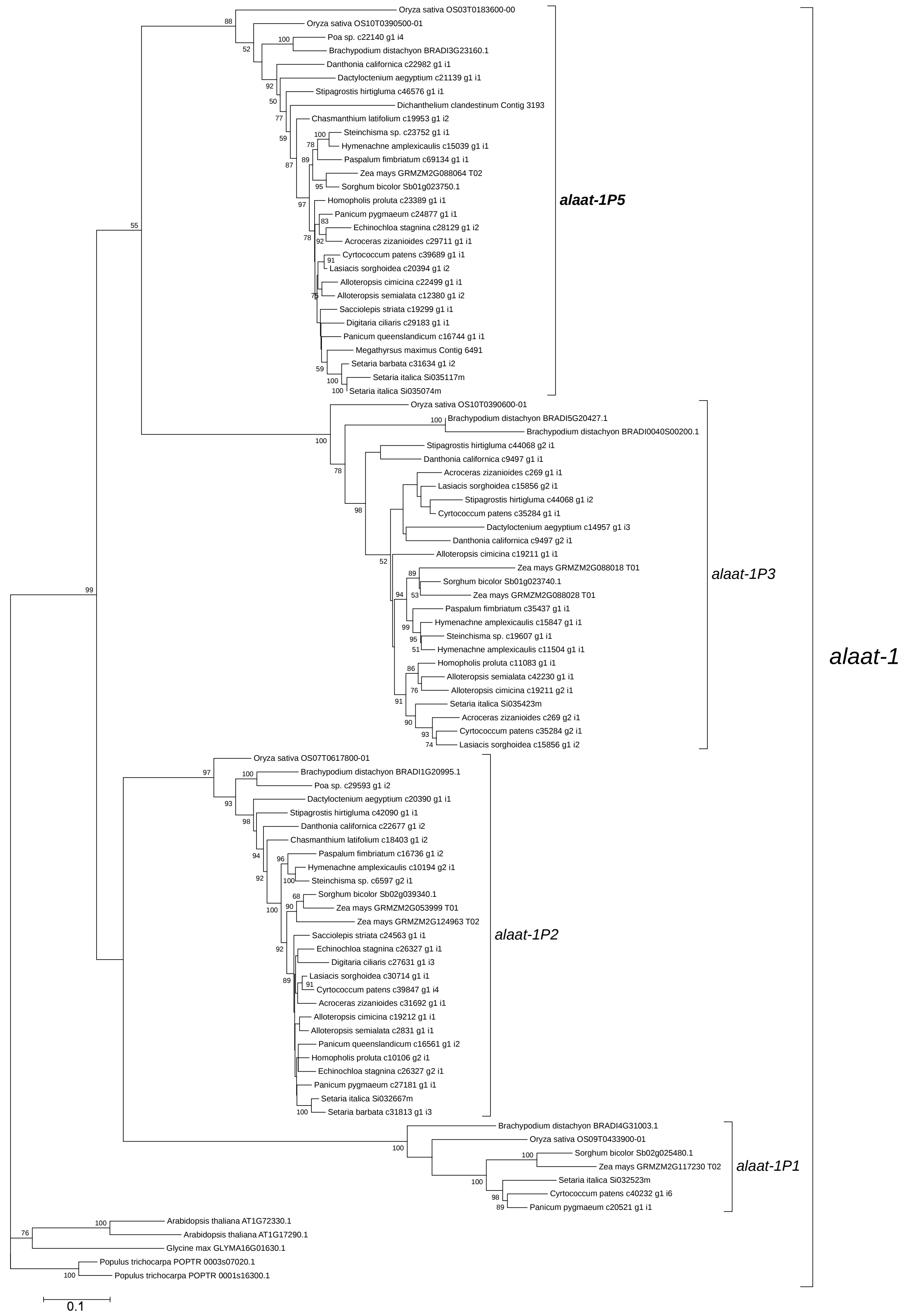

Pyruvate, phosphate dikinase (PPDK)

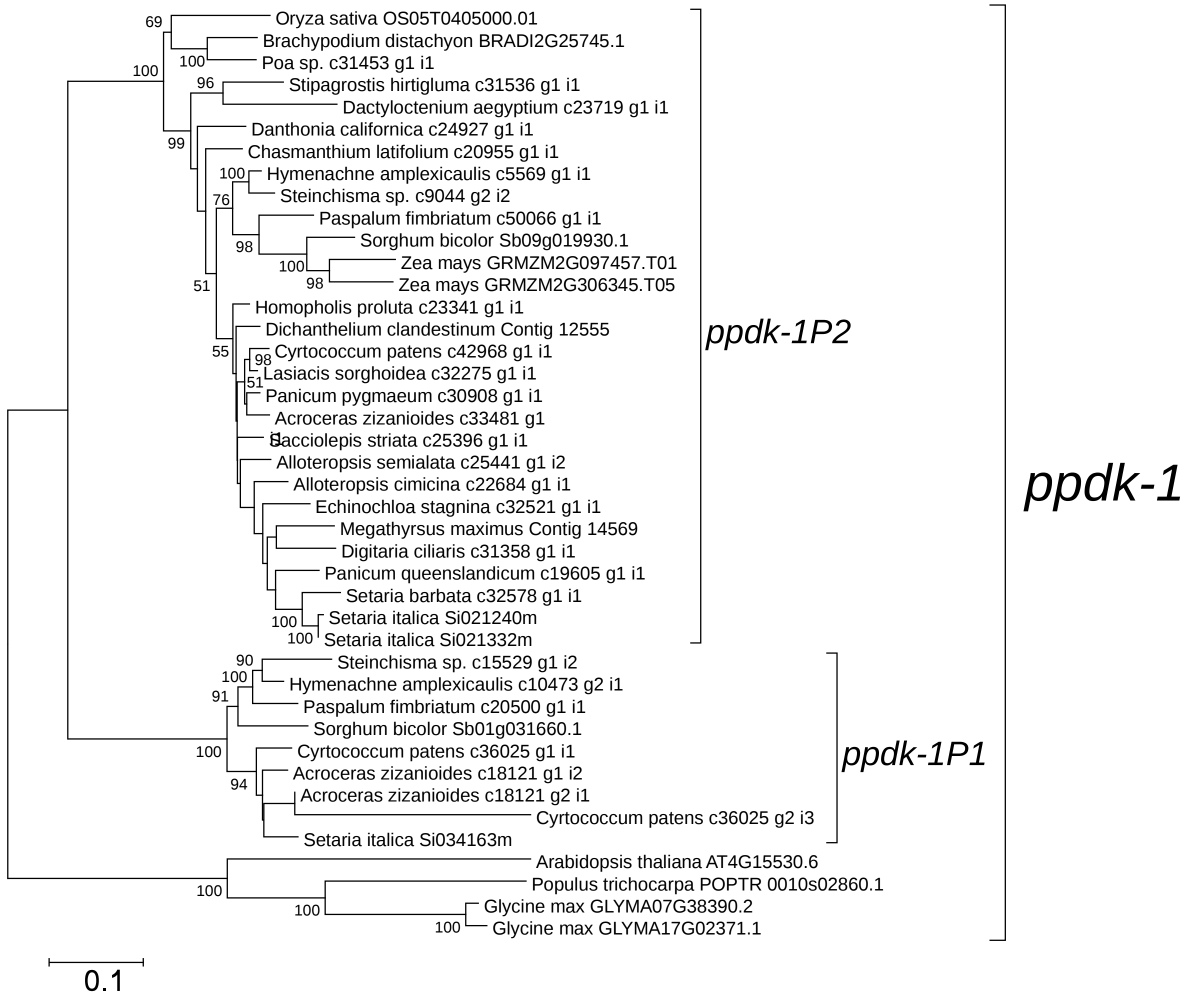

β-carbonic anhydrase (βCA)

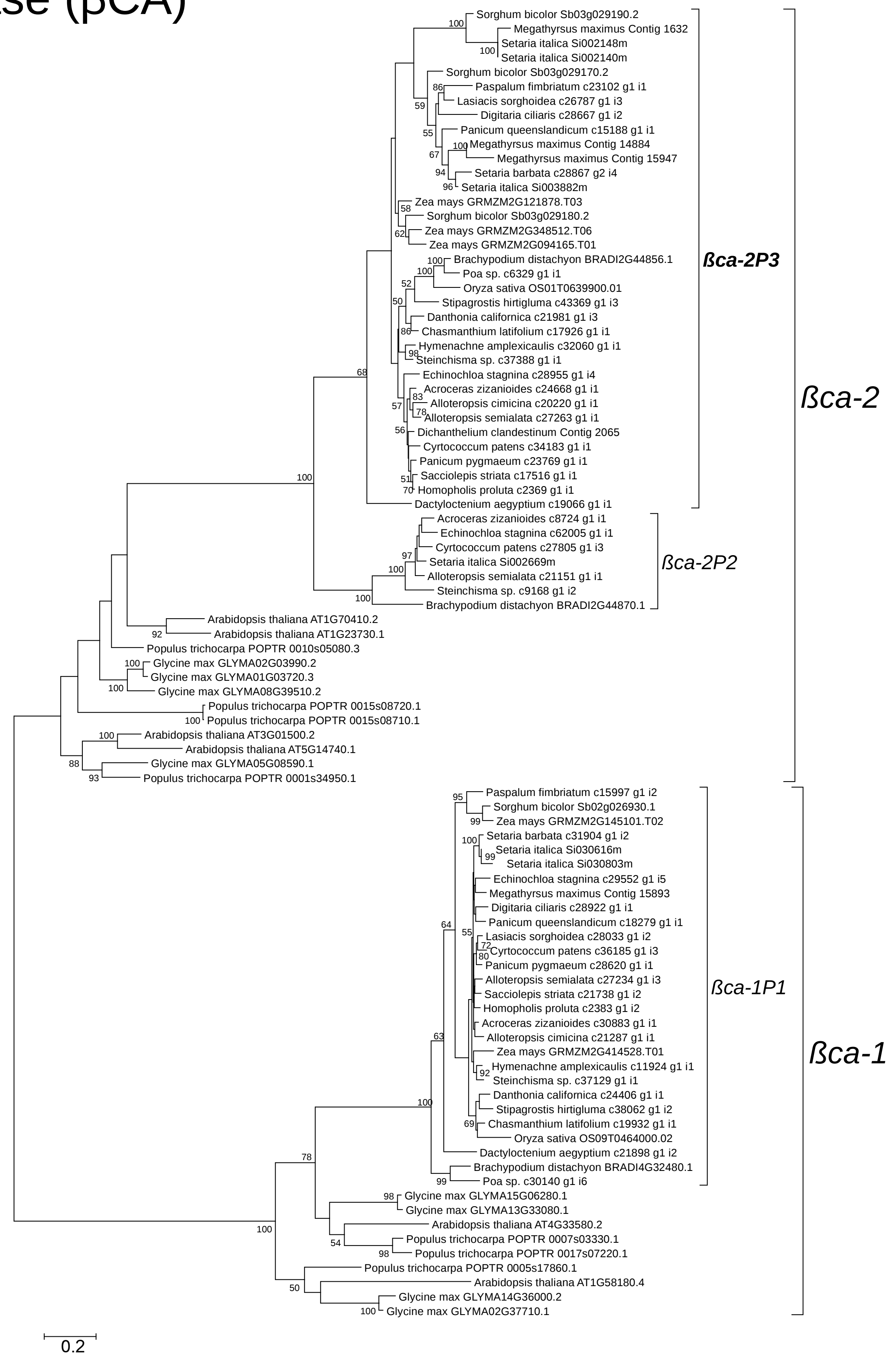

NAD-malate dehydrogenase (NAD-MDH)

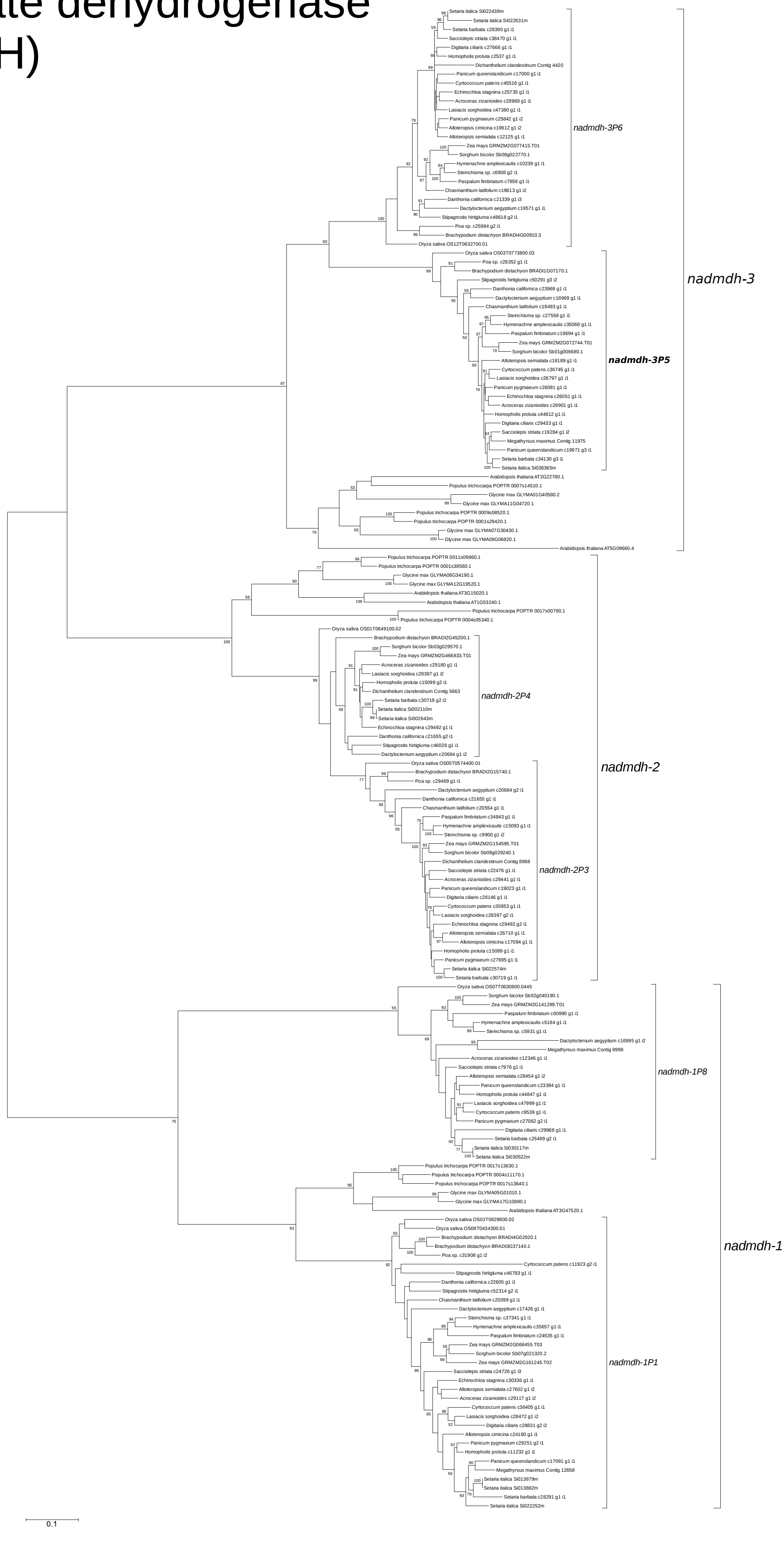

NAD-malate dehydrogenase (NAD-MDH) continuation

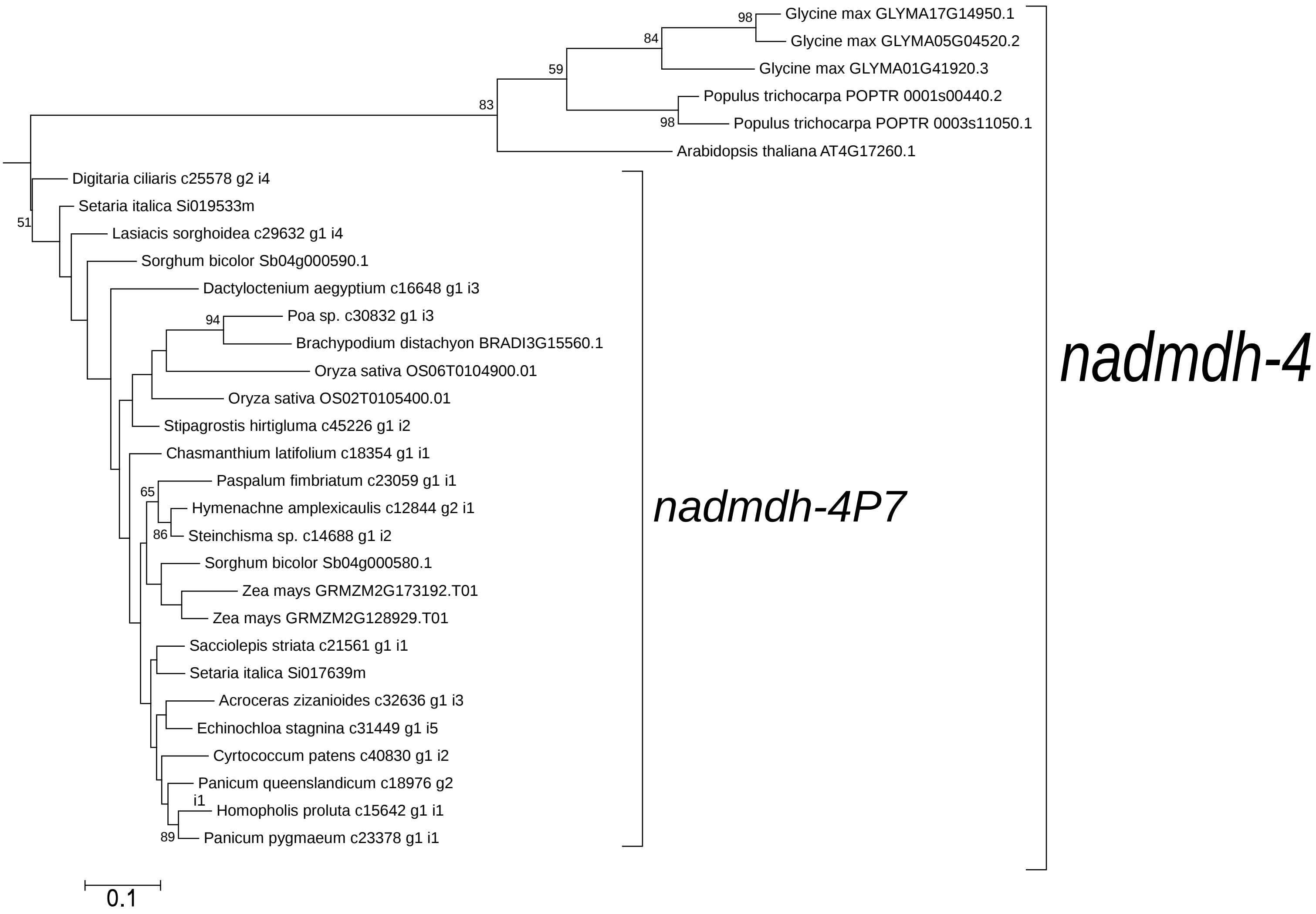

NADP-malic enzyme (NADP-ME)

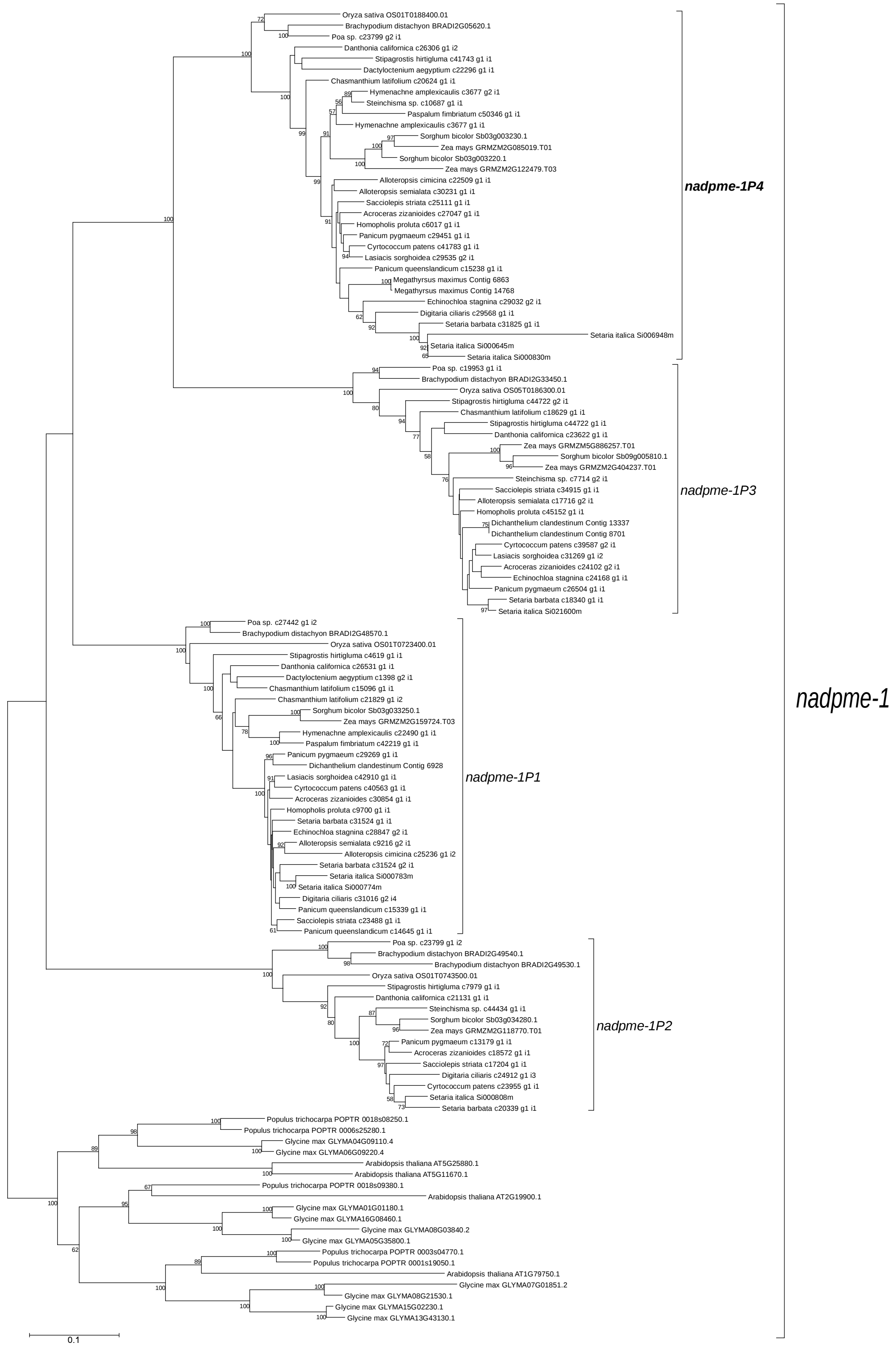

NAD-malic enzyme (NAD-ME)

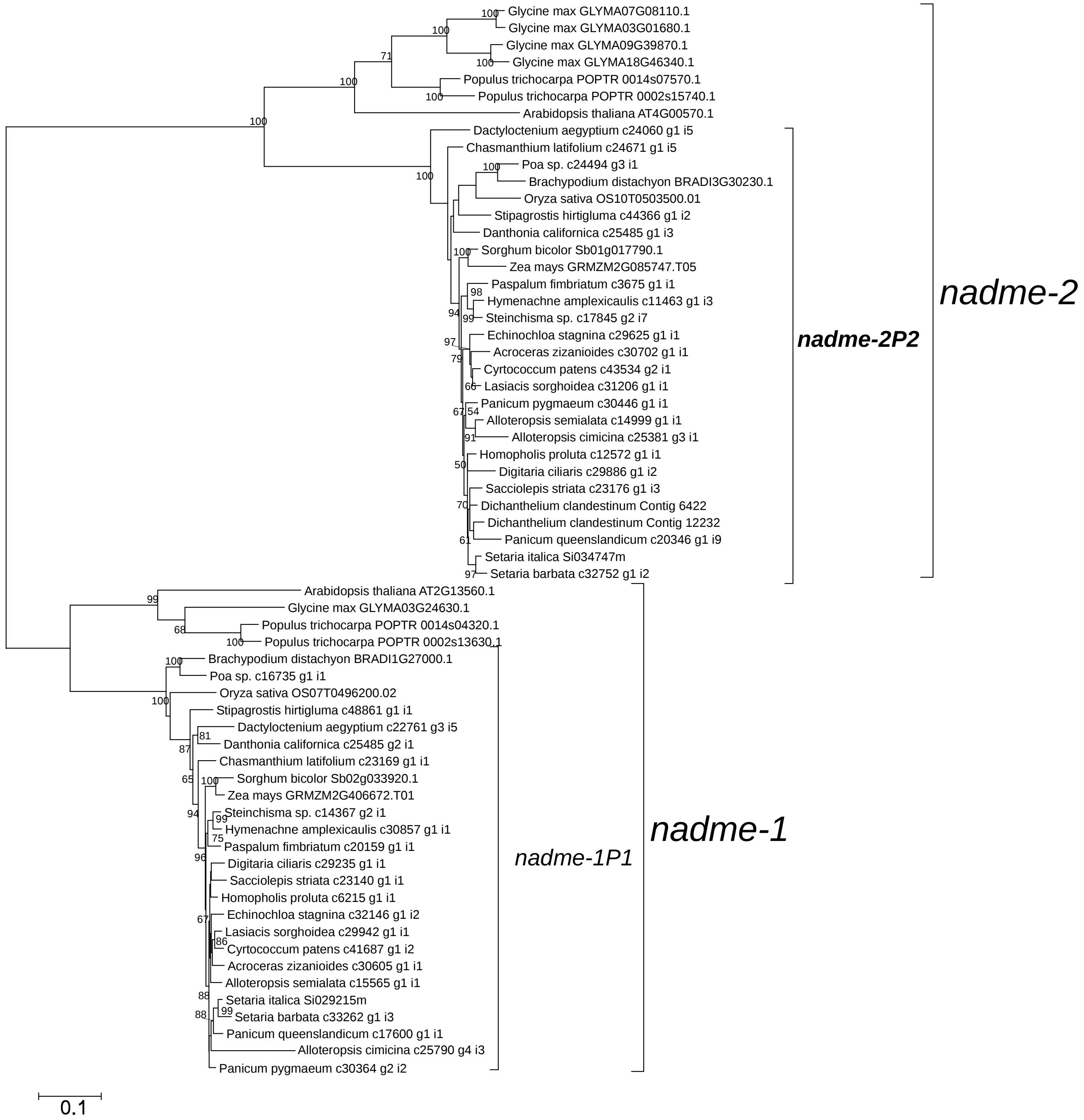

NAD(P)-malate dehydrogenase (NAD(P)-MDH)

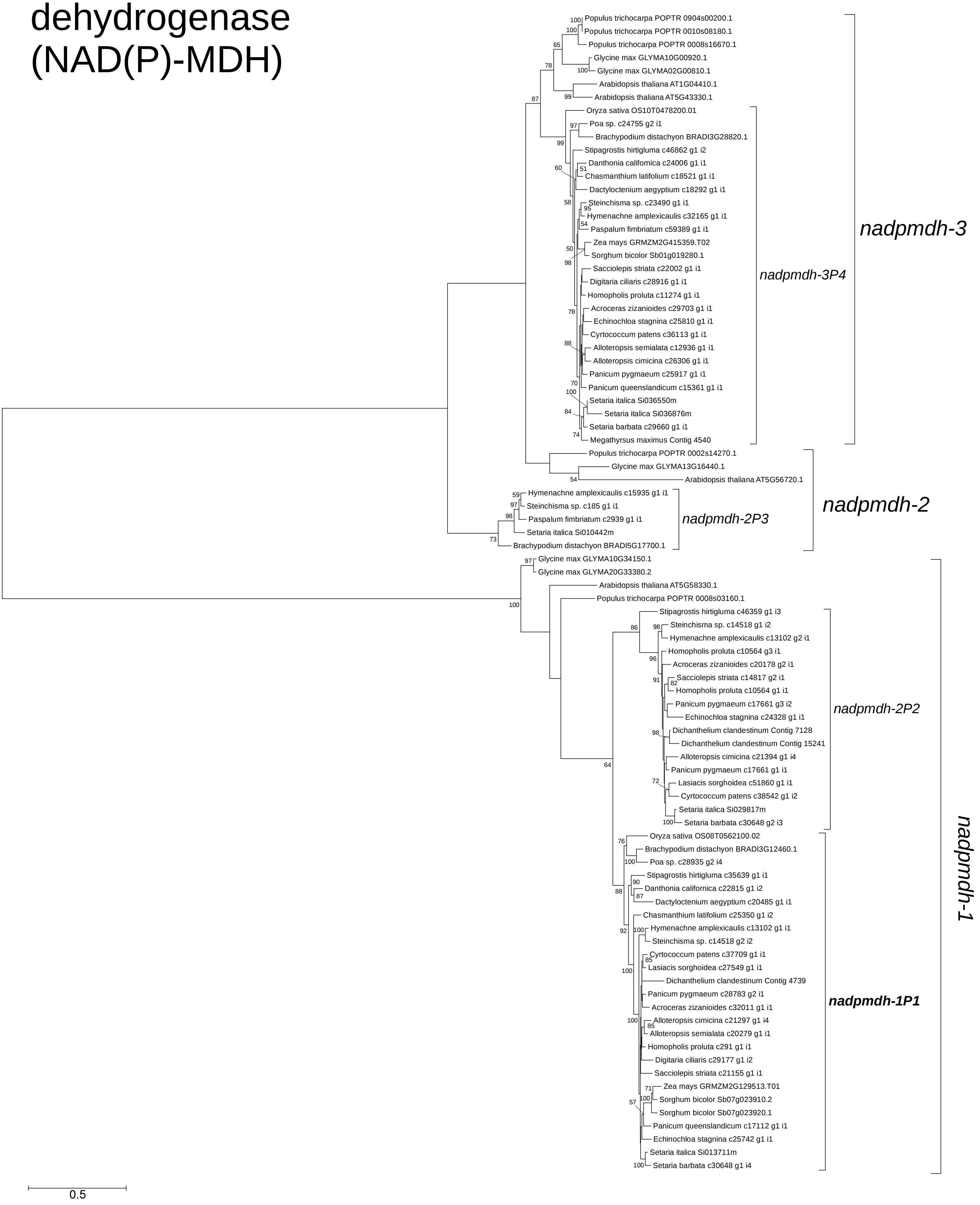

Adenylate kinase protein (AK)

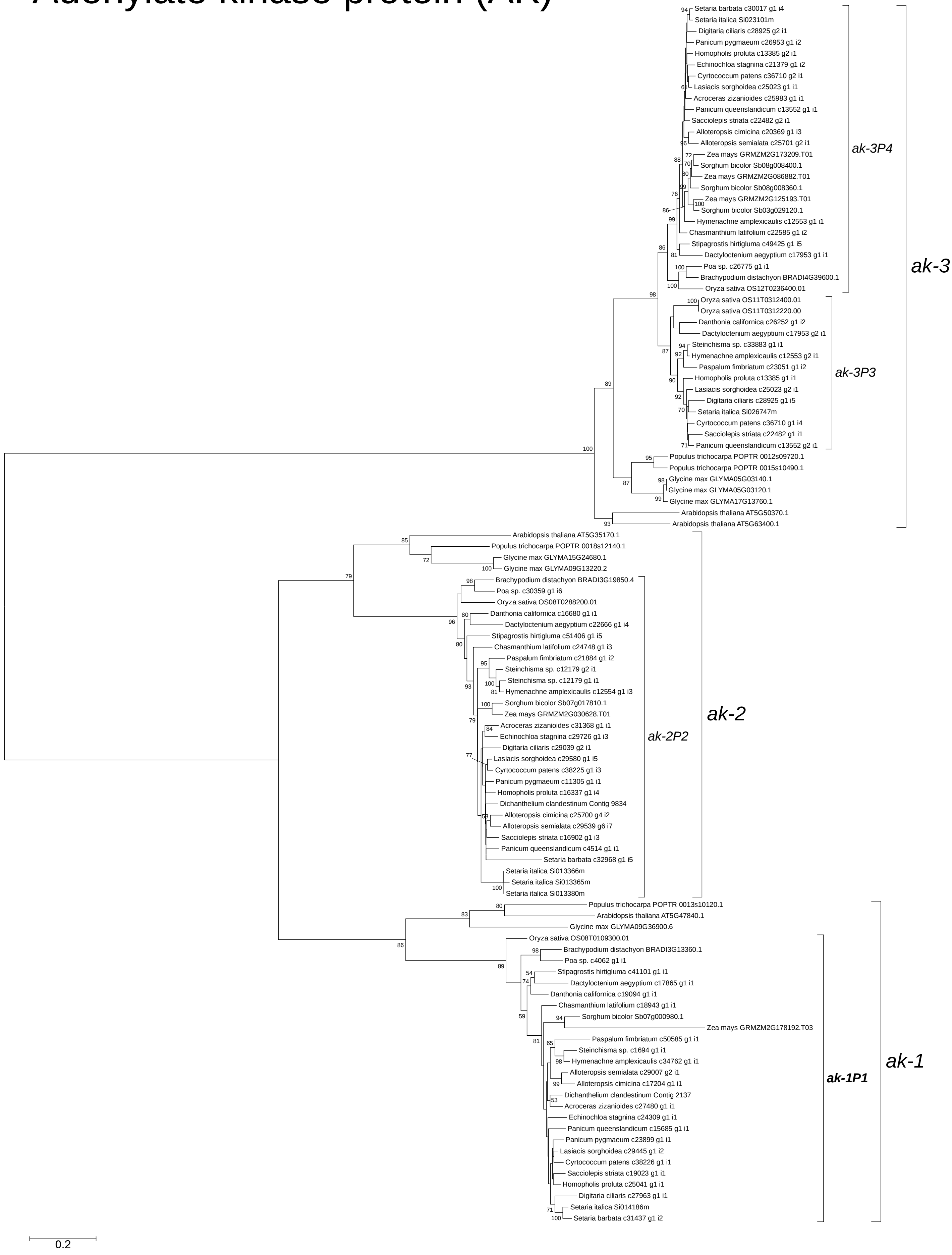

Sodium bile acid symporter family (SBAS)

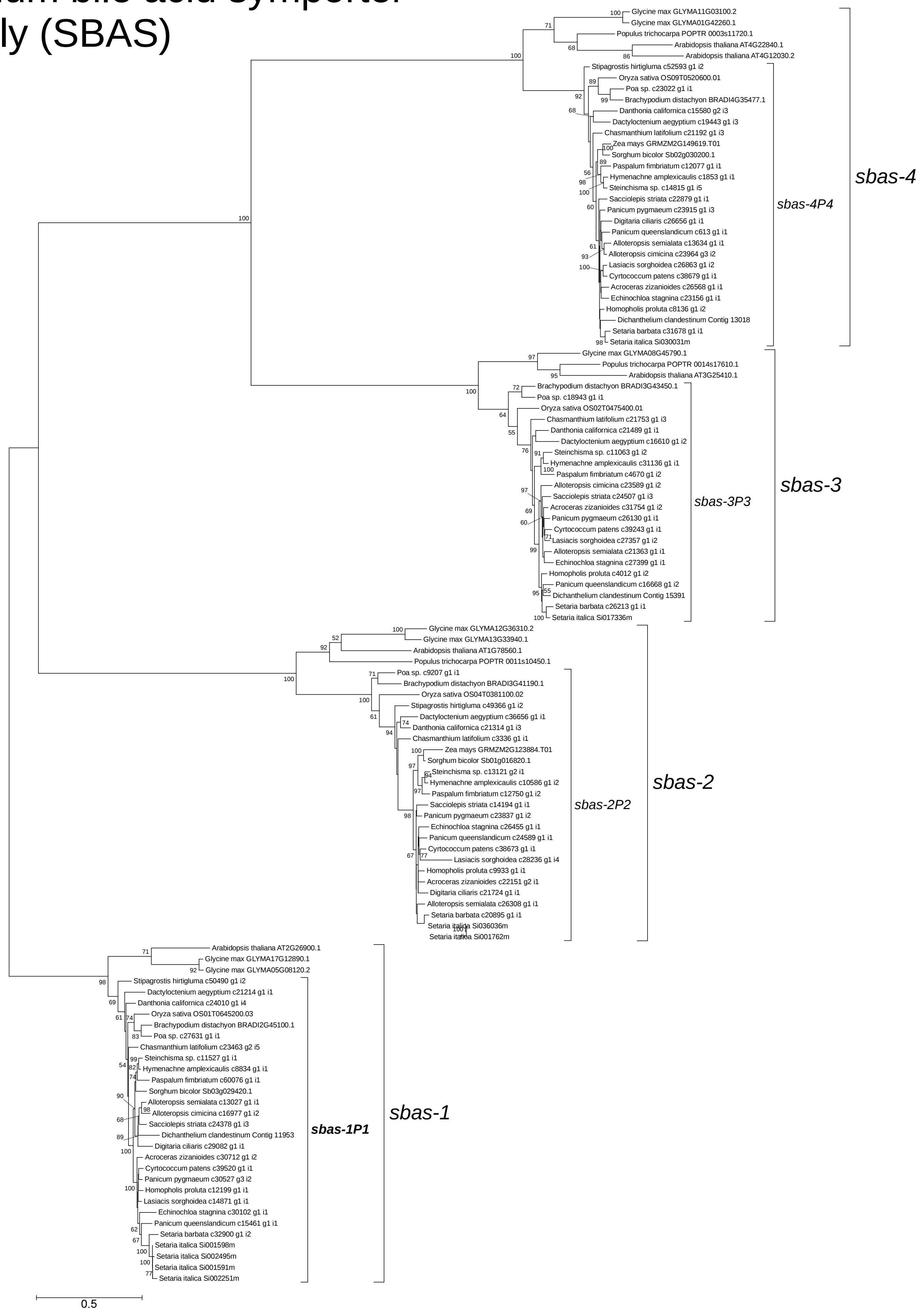

Dicarboxylate carrier (DIC)

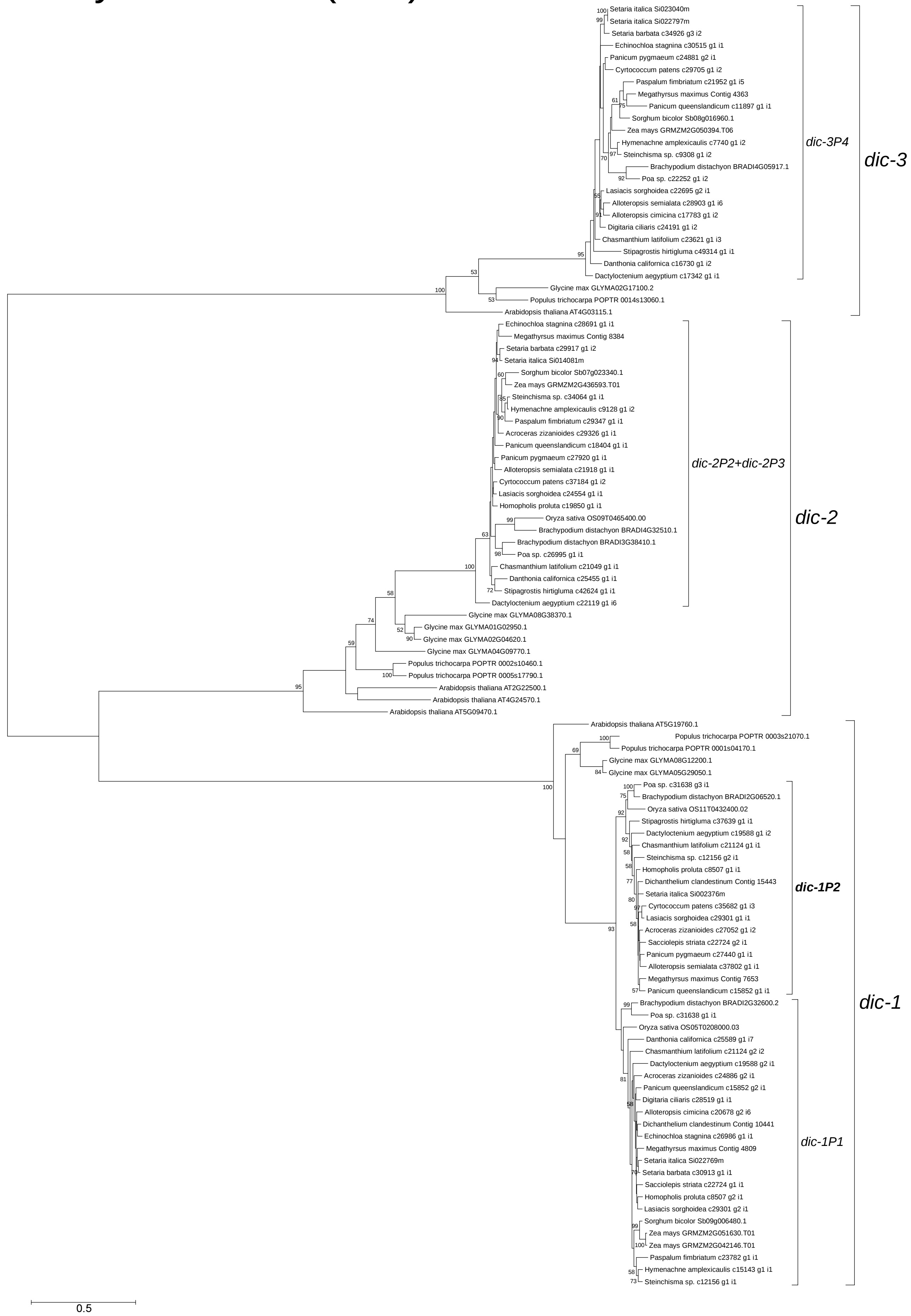

Dicarboxylate transporter (DIT)

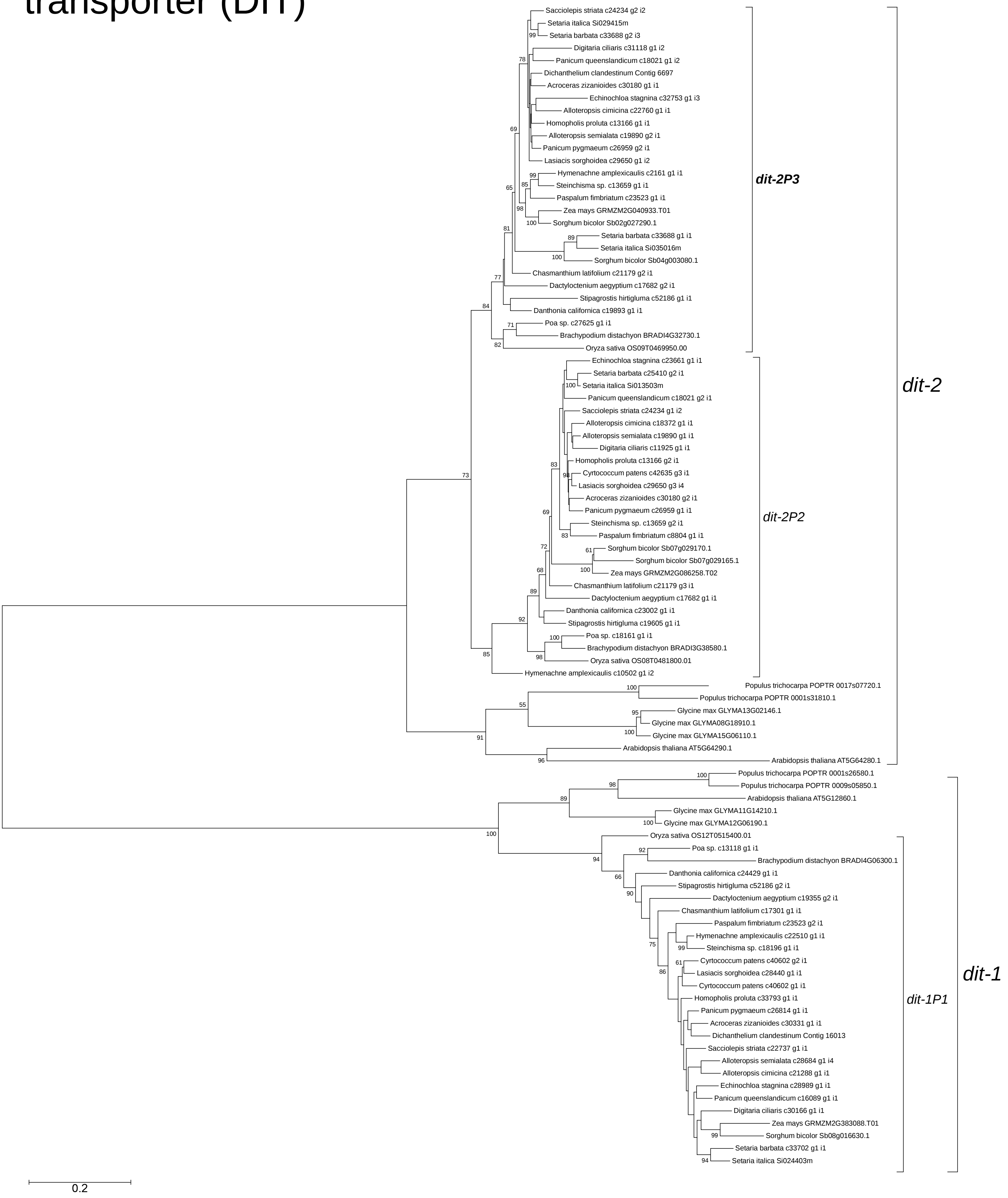

Glyceraldehyde-3-phosphate Dehydrogenase (GAPDH)

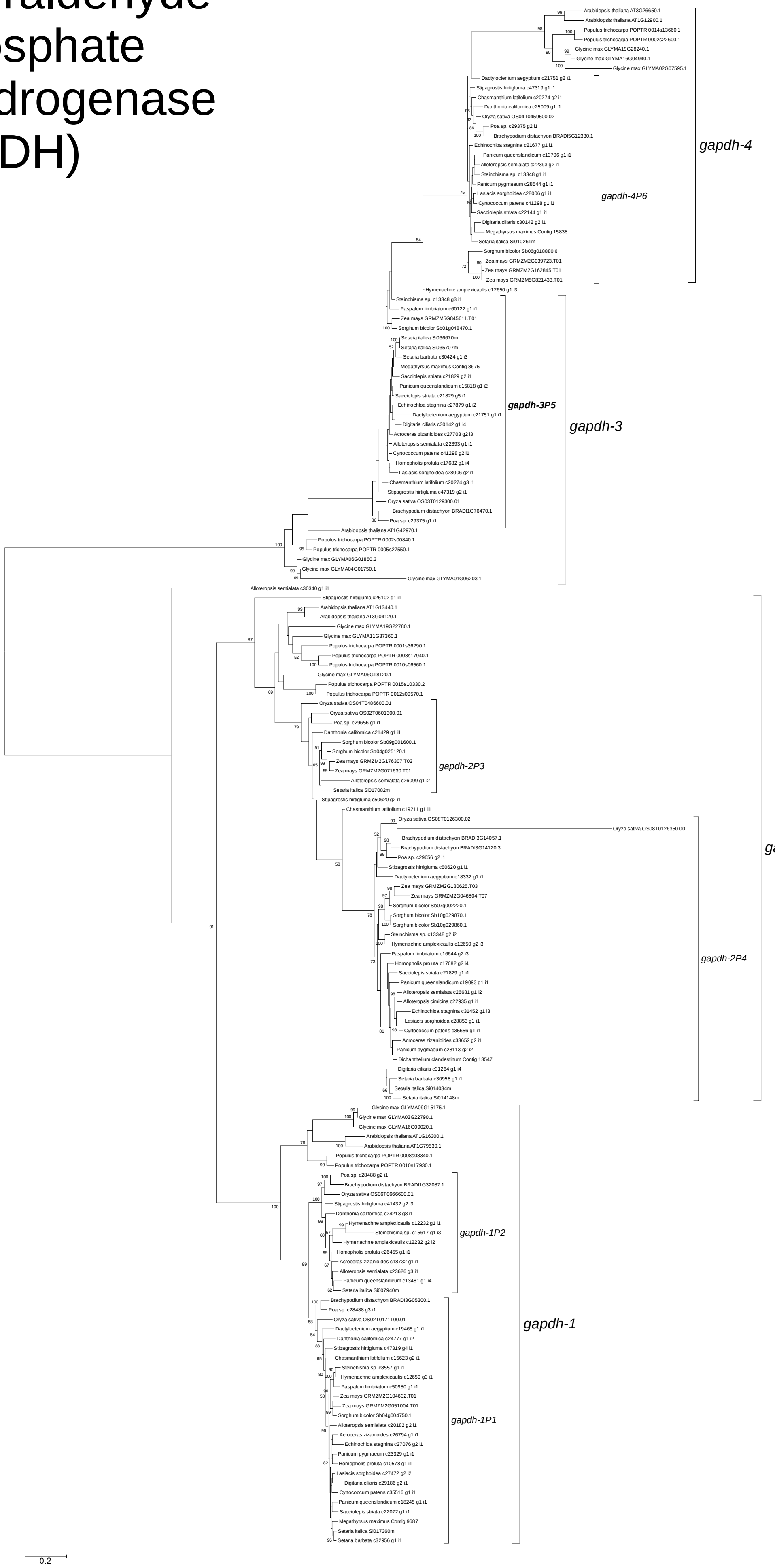

Sodium:Hydrogen antiporter (NHD)

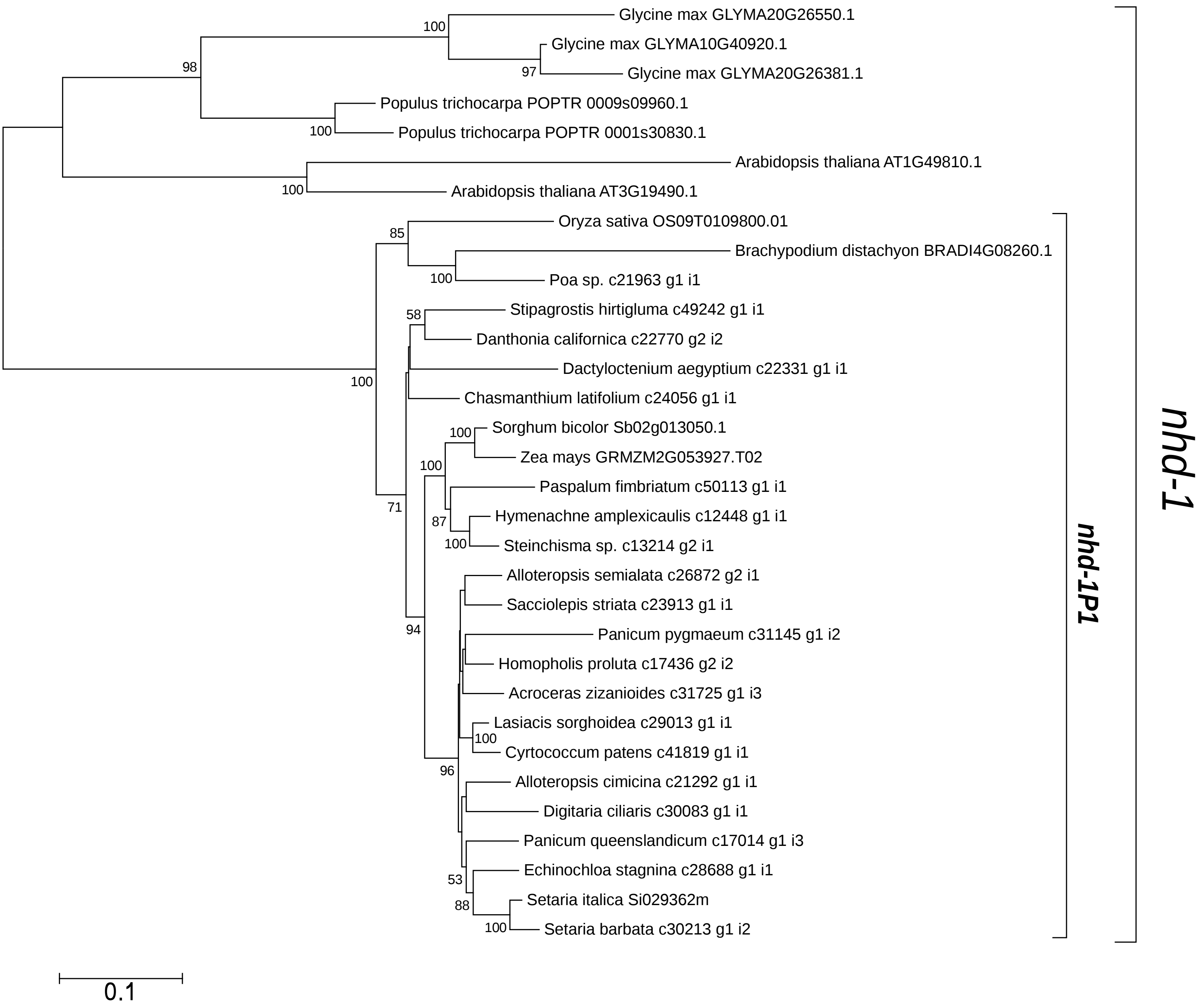

Phosphoenolpyruvate carboxylase kinase (PEPC-K)

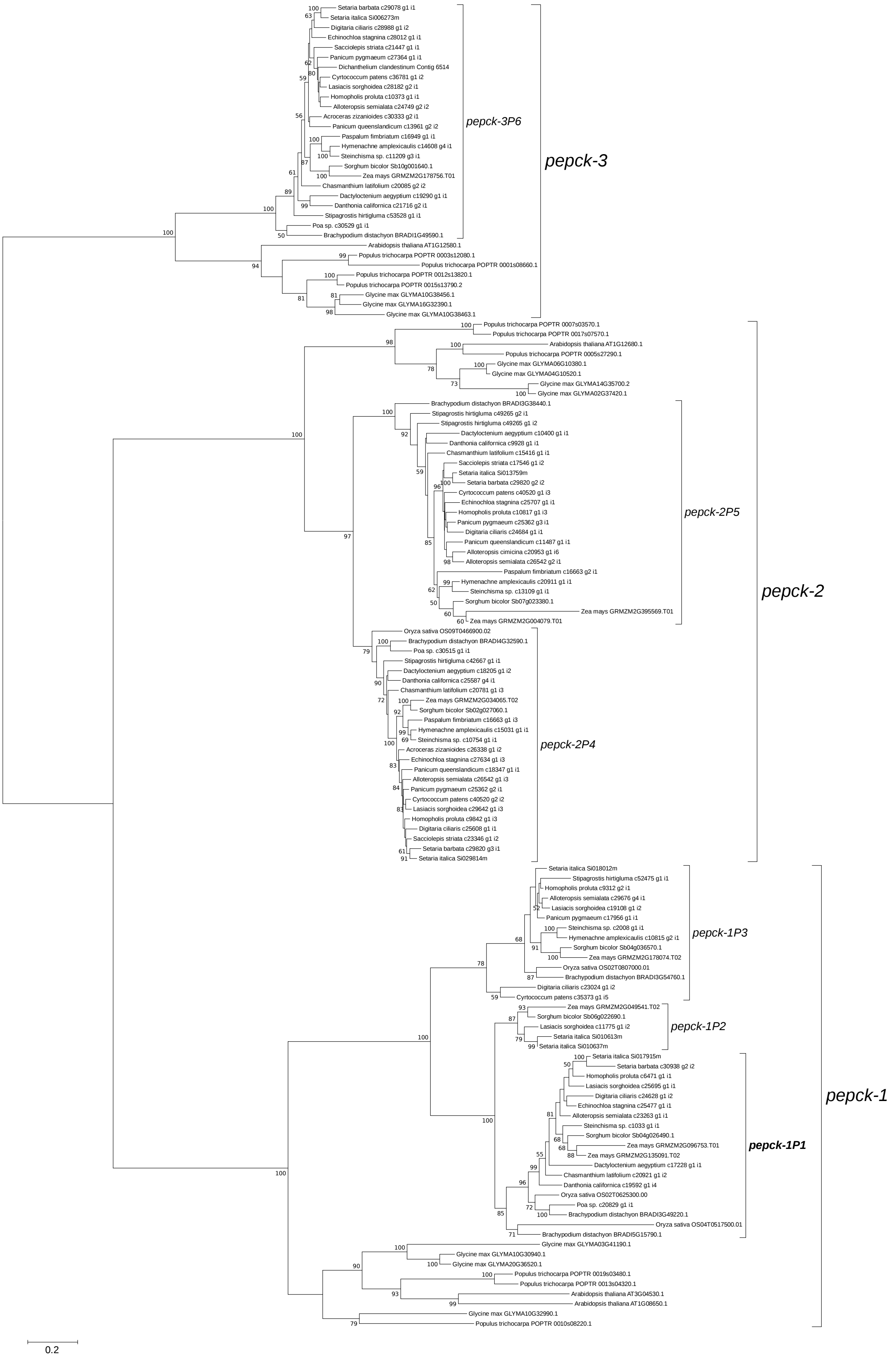

Phosphoglycerate Kinase (PGK)

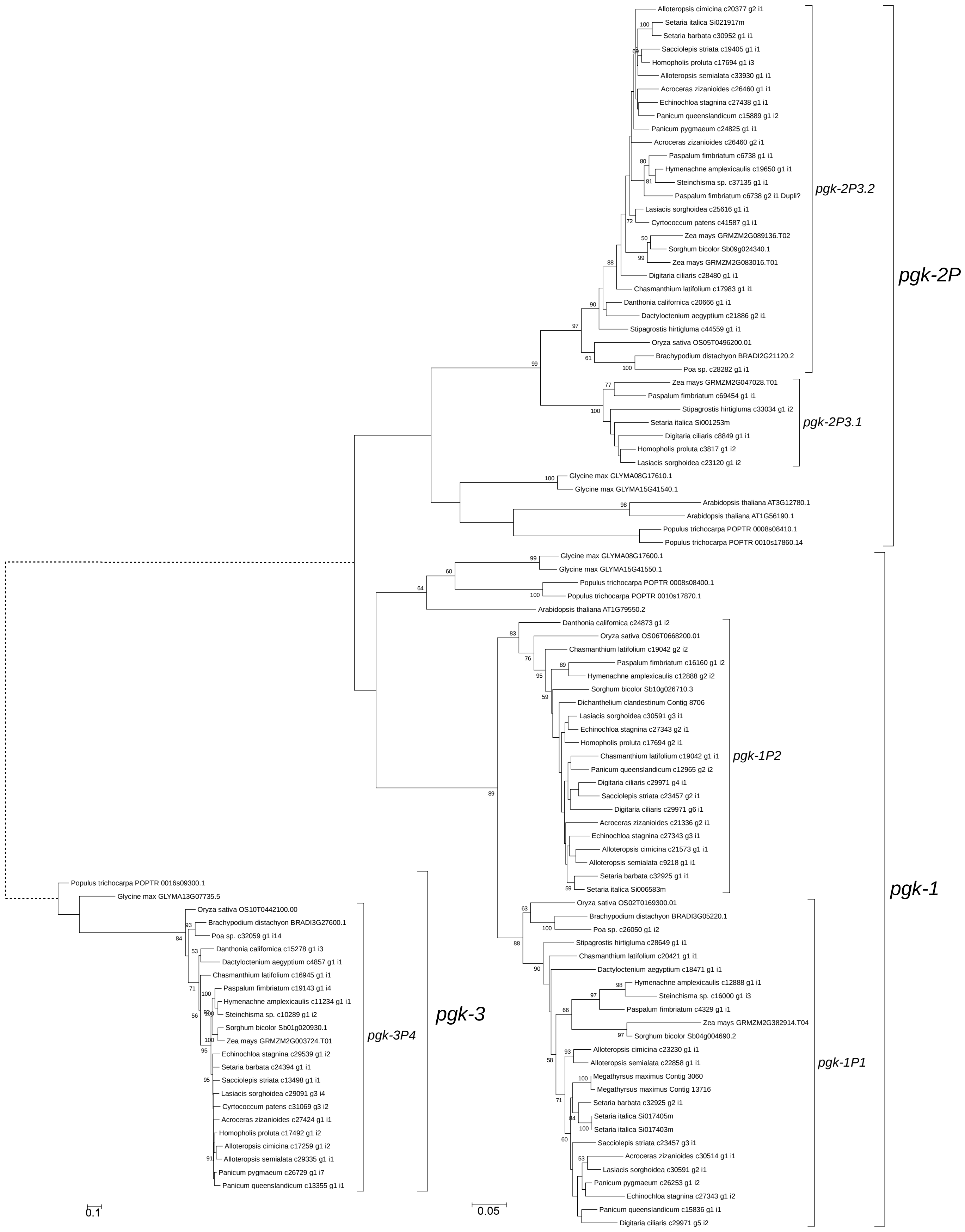

PYRUVATE KINASE (PK)

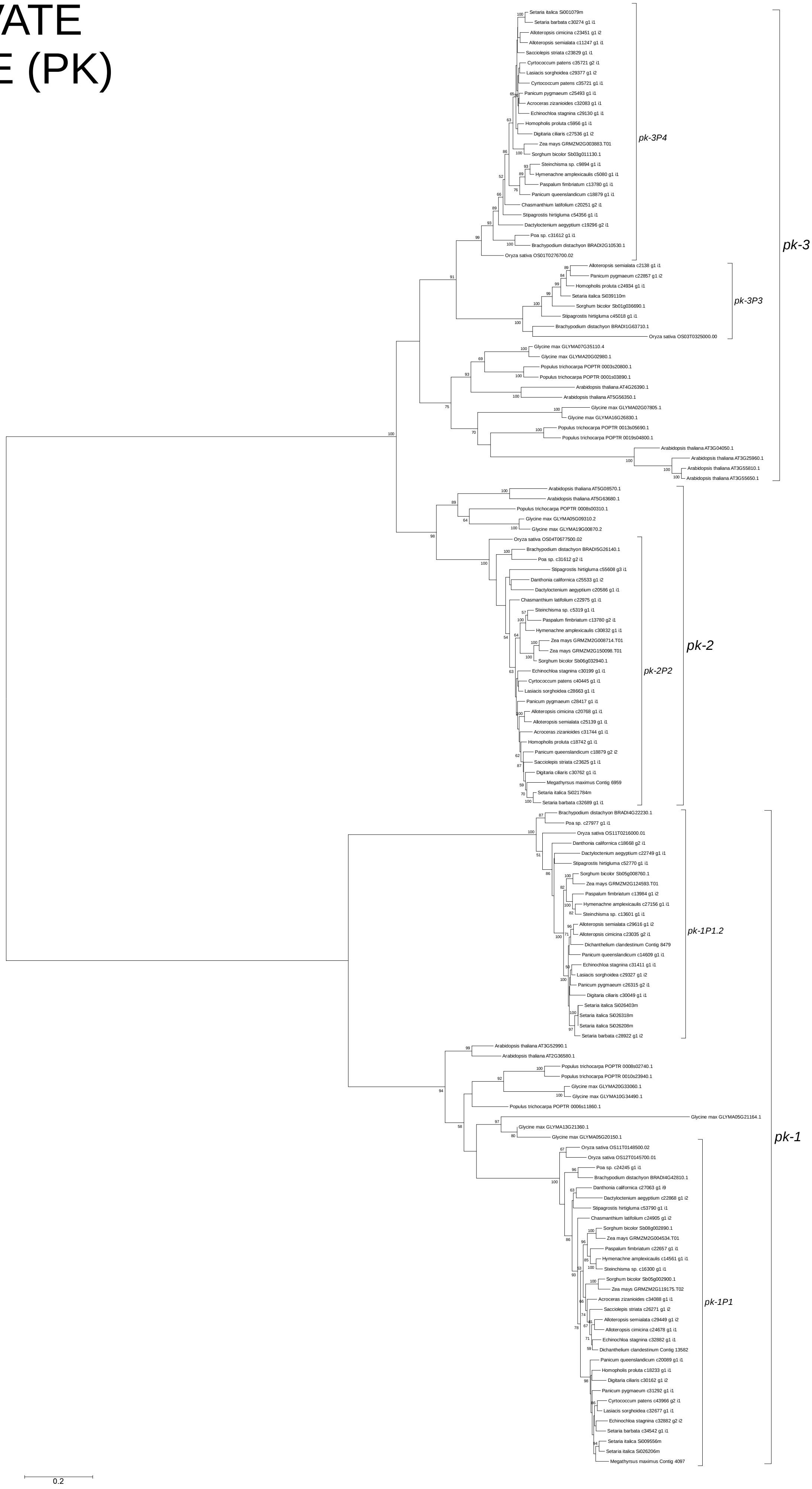

Inorganic pyrophosphatase (PPa)

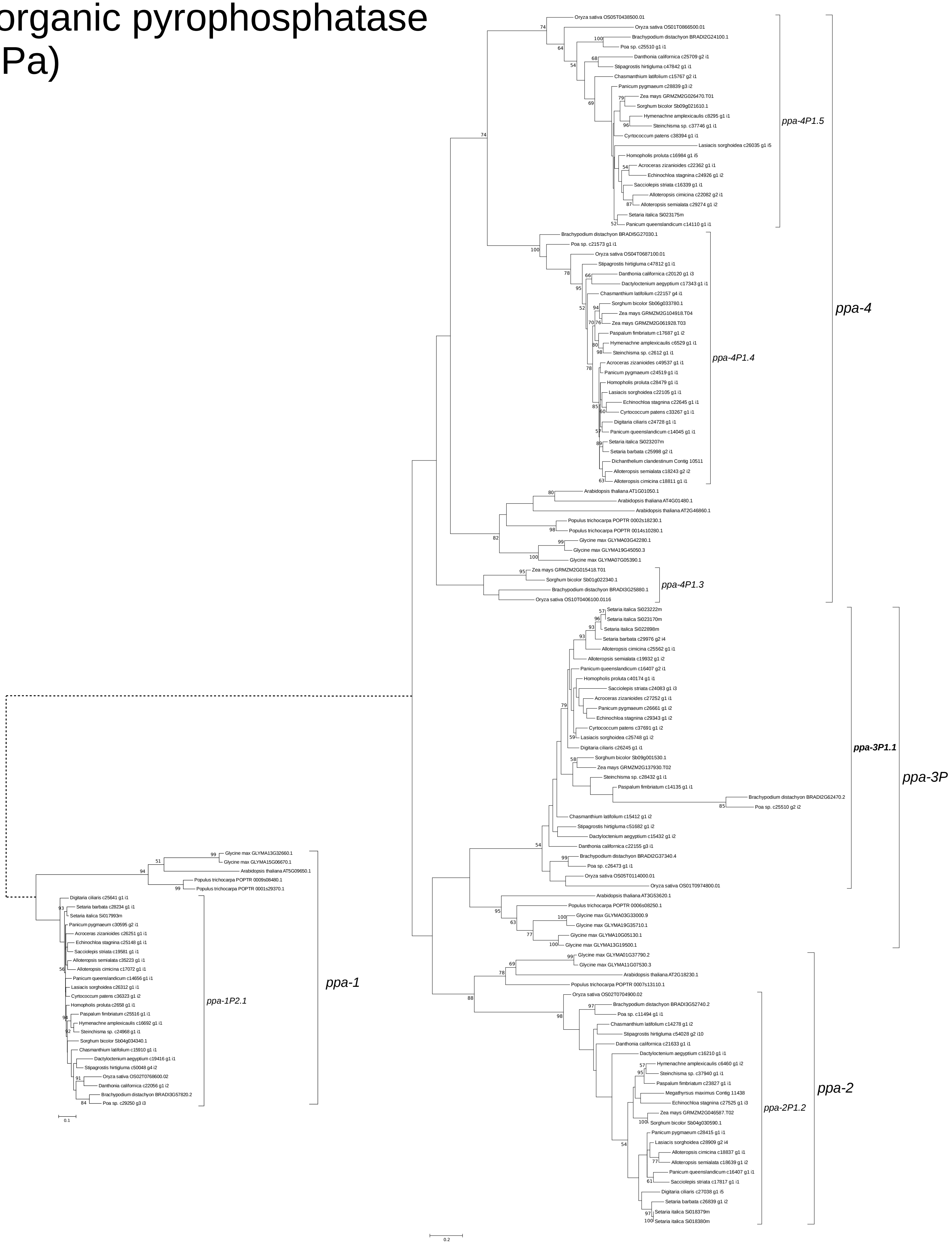

Pyruvate, phosphate dikinase regulatory protein (PPDK-RP)

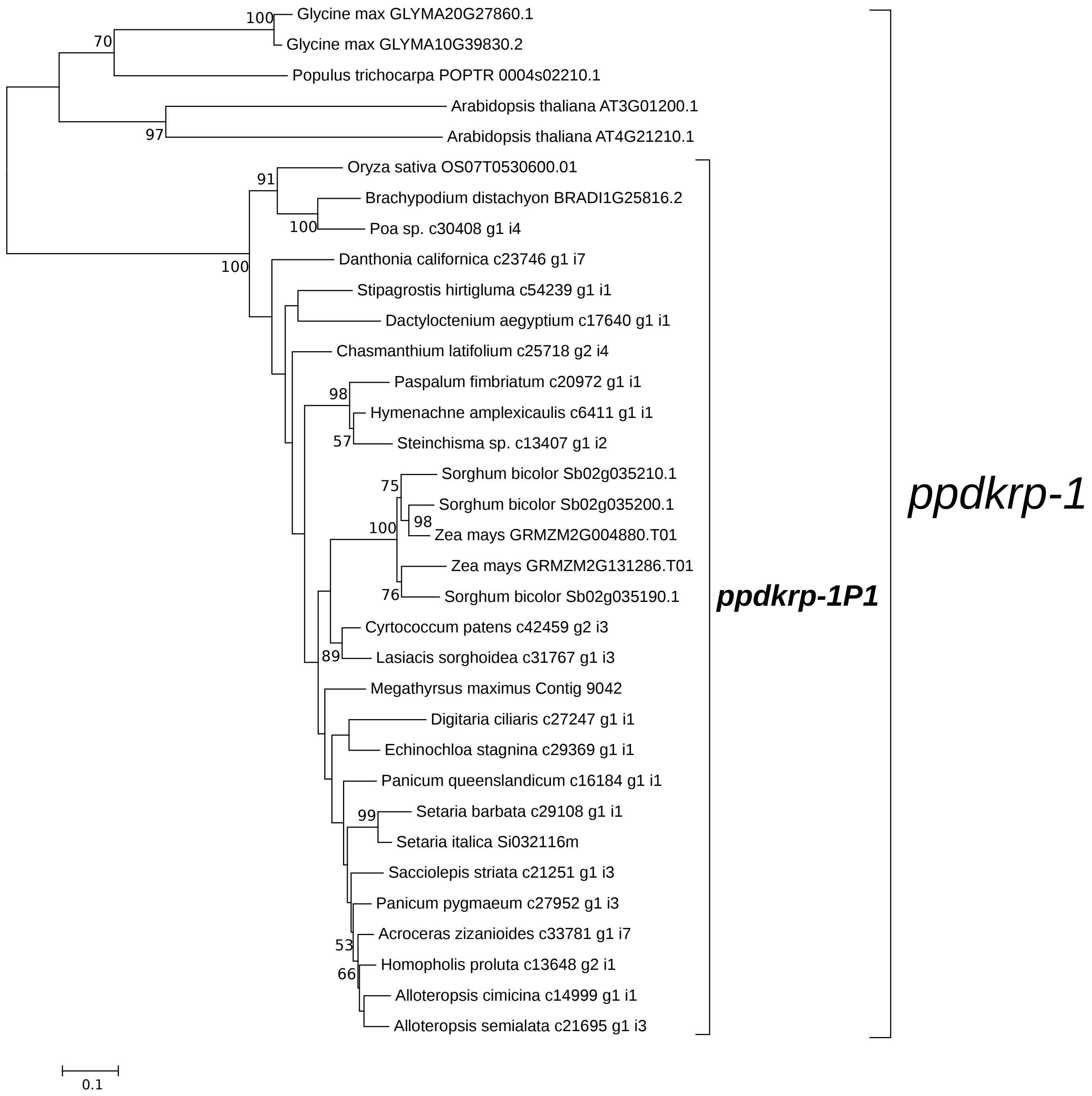

Phosphoenolpyruvate-phosphate translocator (PPT)/ Triosephosphate-phosphate translocator (TPT)/ Glucose-6-phosphate/phosphate translocator (GPT)

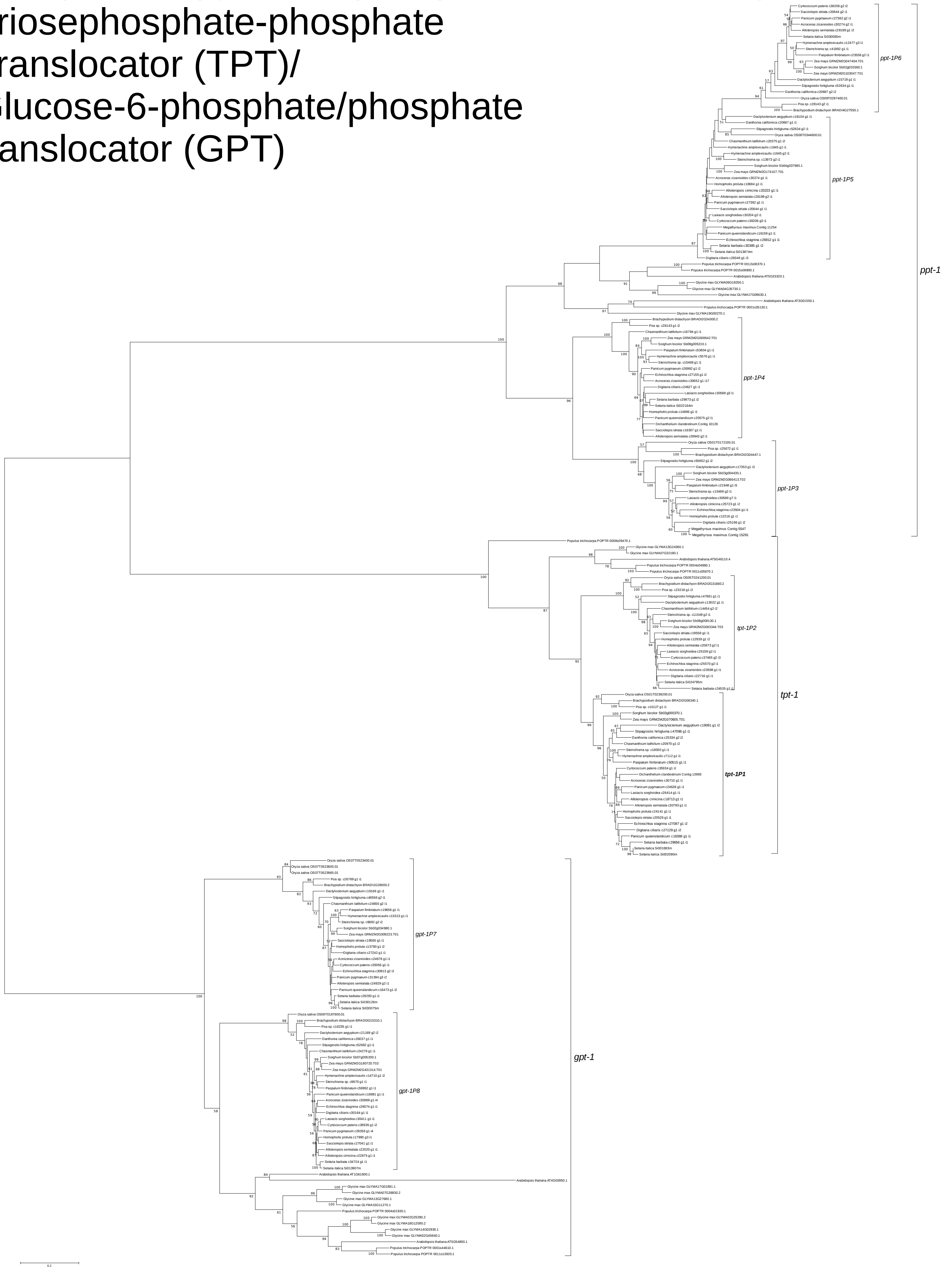

Tonoplast malate/fumarate transporter (TDT)

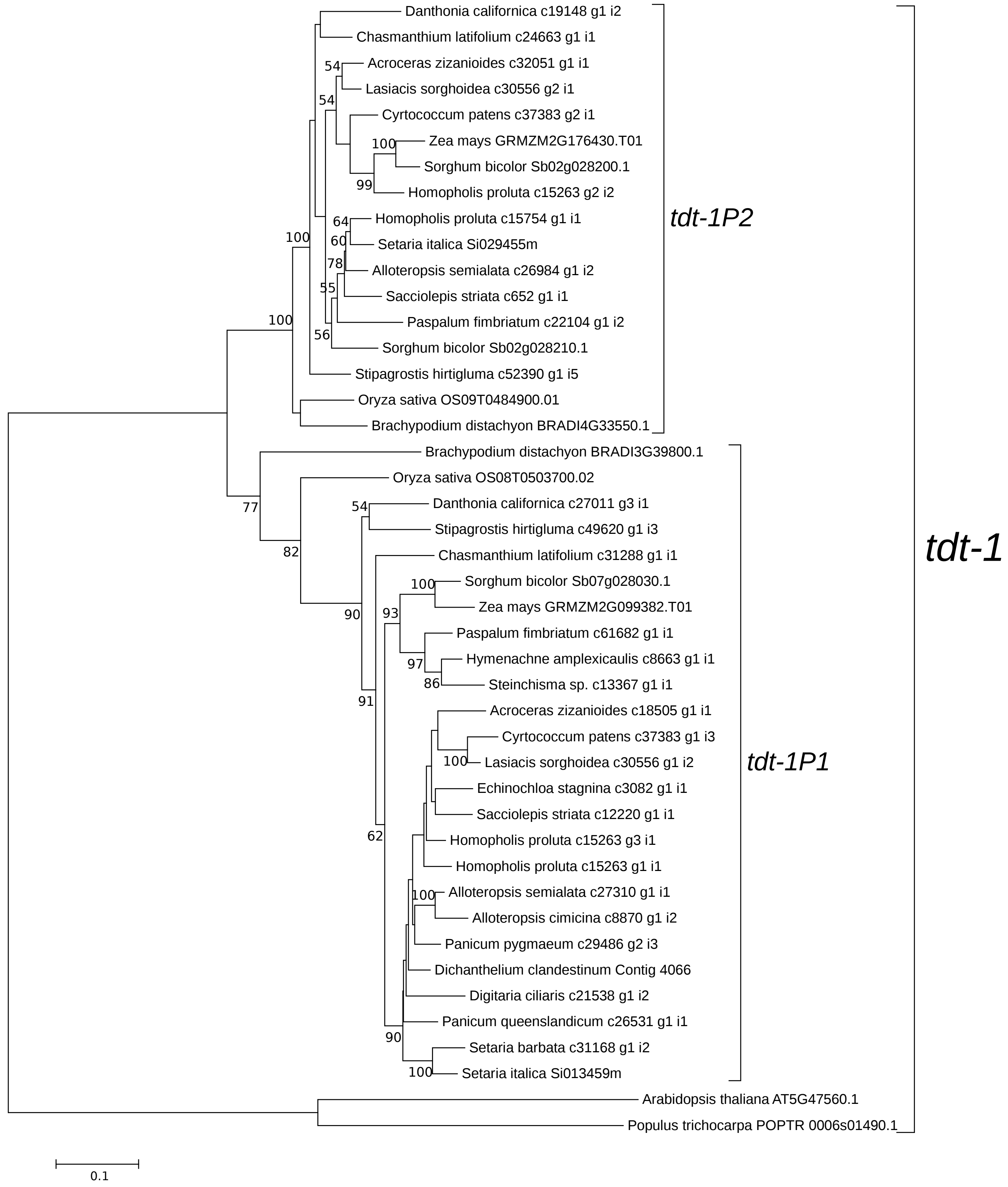

Serine--glyoxylate aminotransferase / Alanine--glyoxylate aminotransferase (AGT)

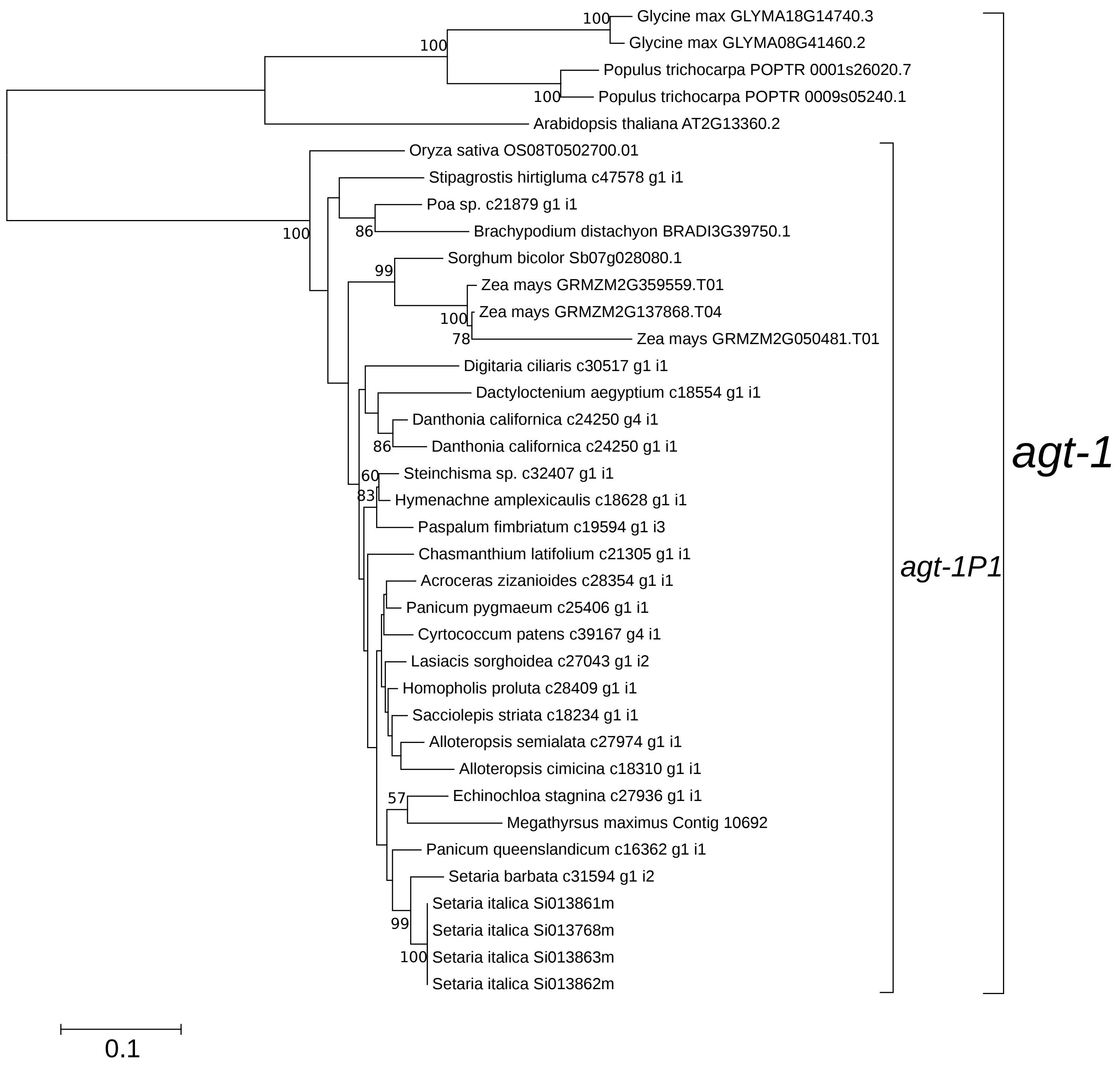

Glycine cleavage system T protein (GCVT)

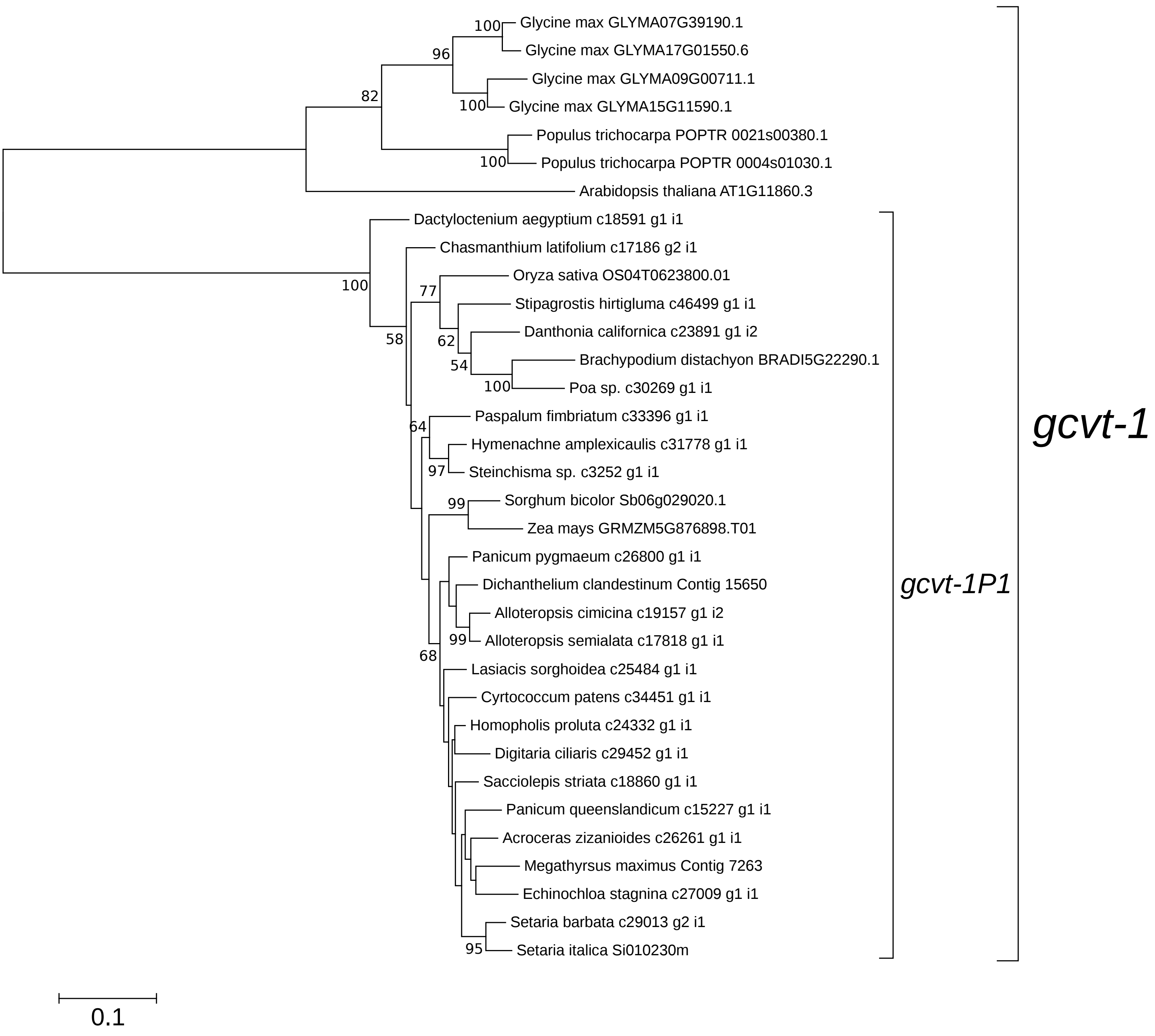

Glycine cleavage system H protein, mitochondrial (GDH)

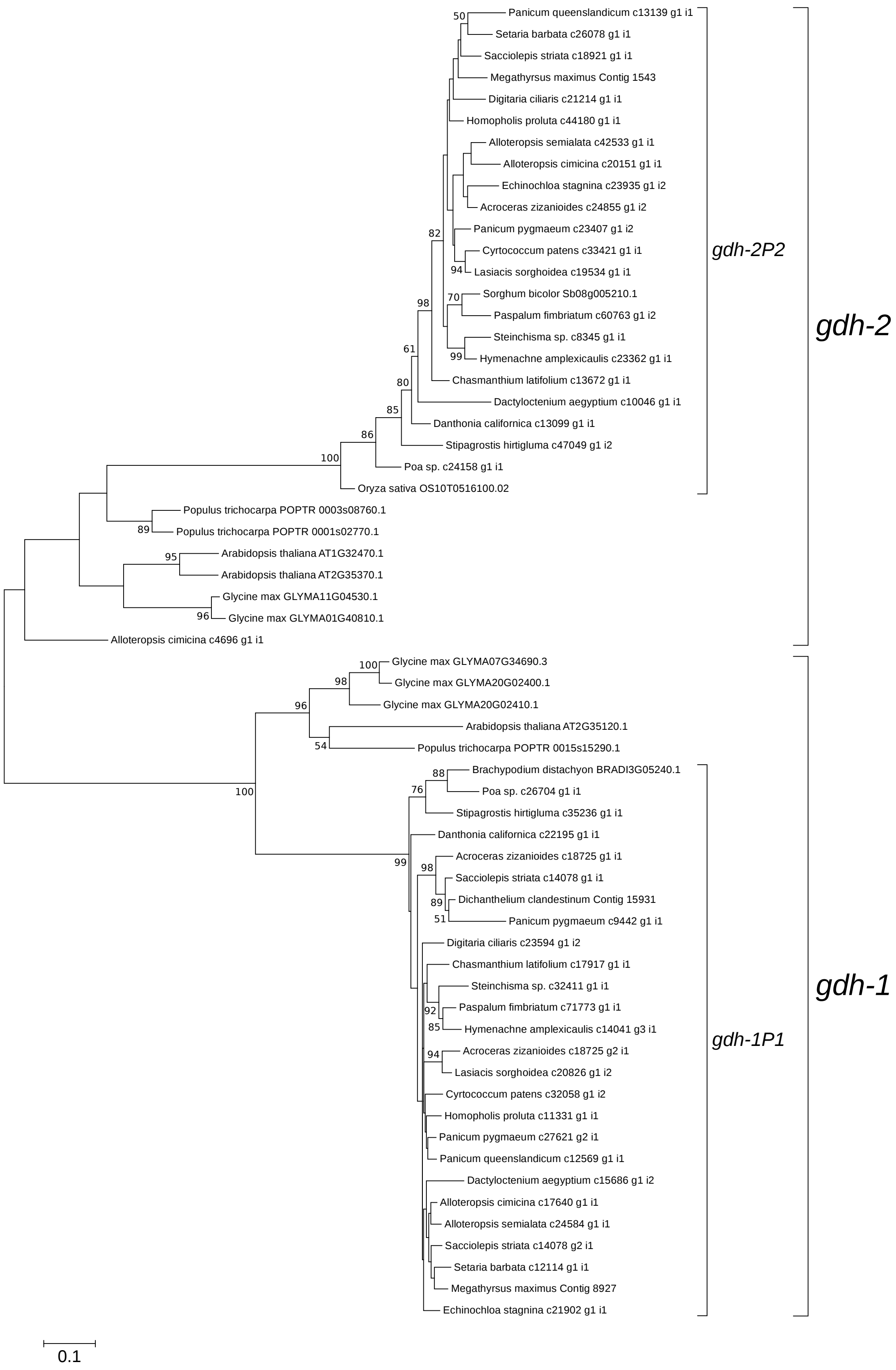

Glutamate--glyoxylate aminotransferase (GGAT)

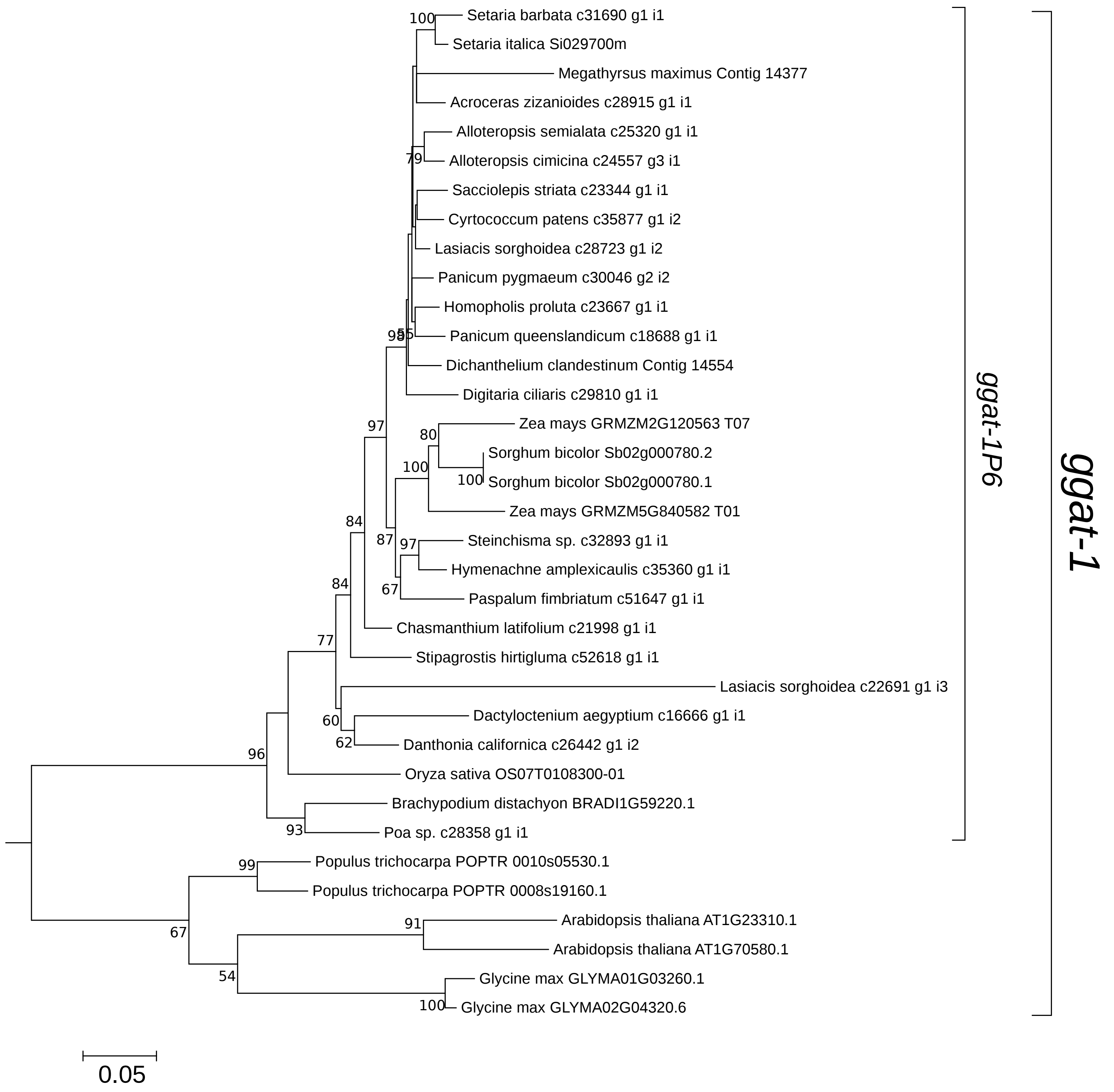

Glycine cleavage system P protein, mitochondrial (GLDP)

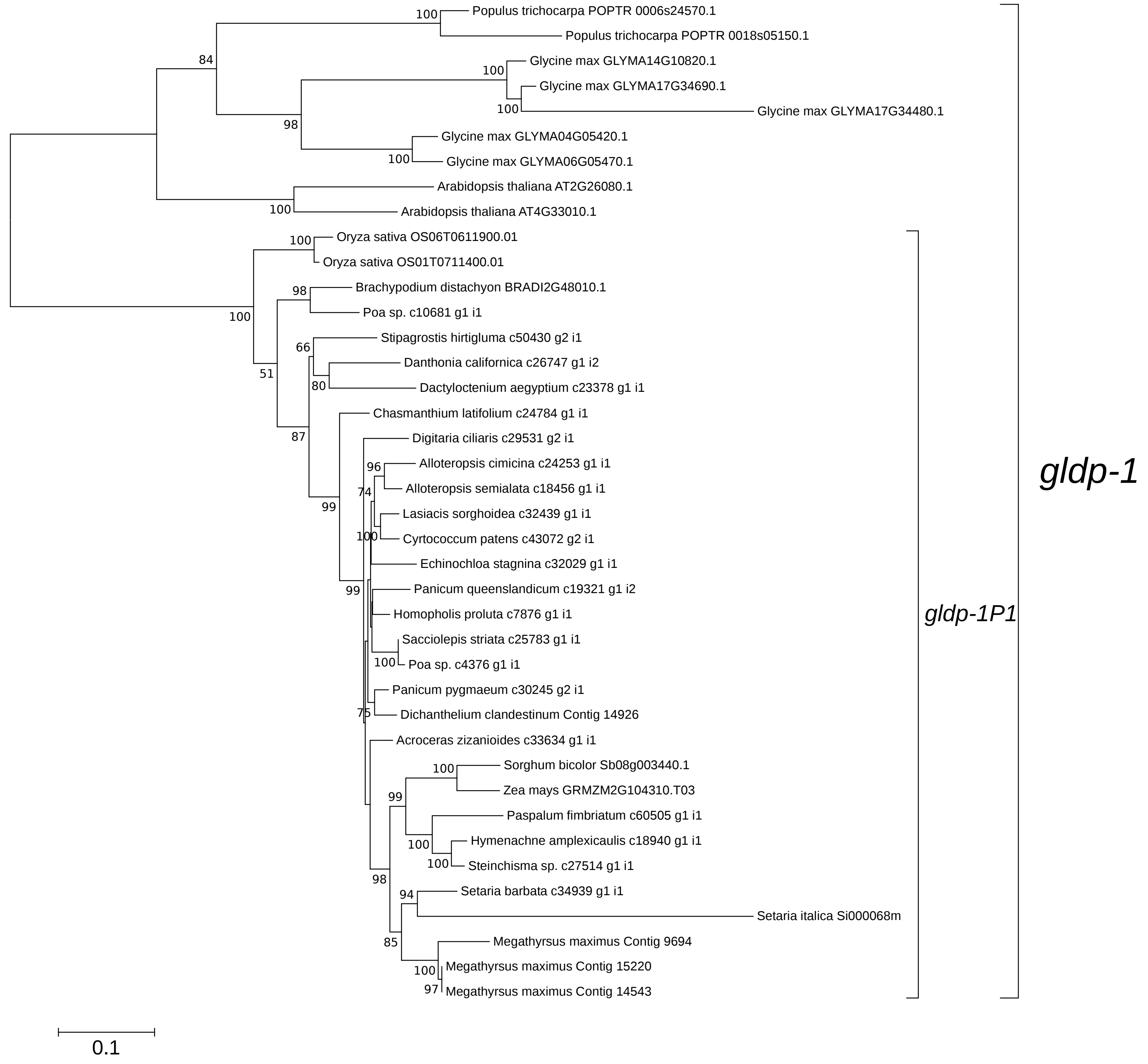

Peroxisomal (S)-2-hydroxy-acid oxidase (GLO)

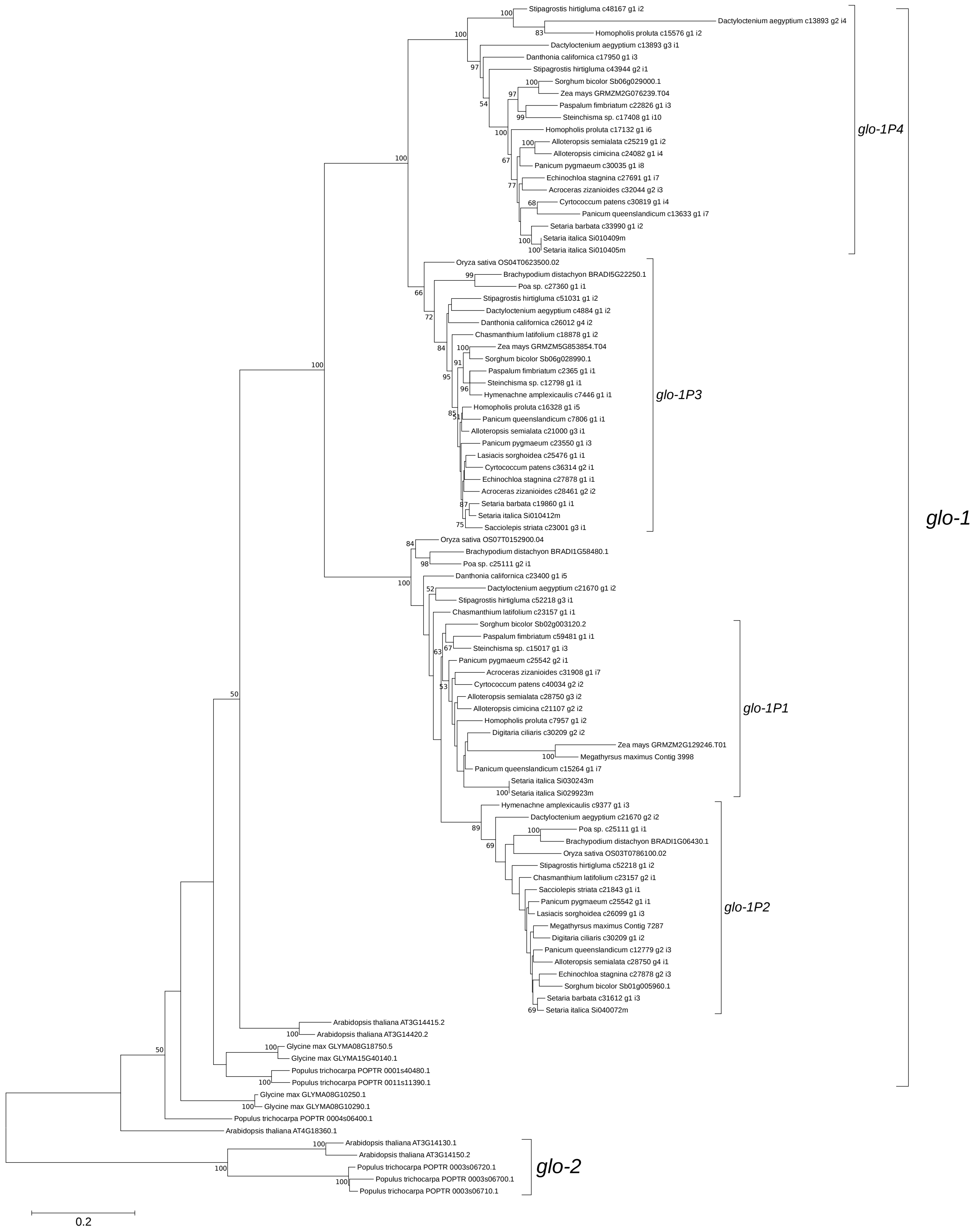

D-glycerate 3-kinase, chloroplastic (GLYK)

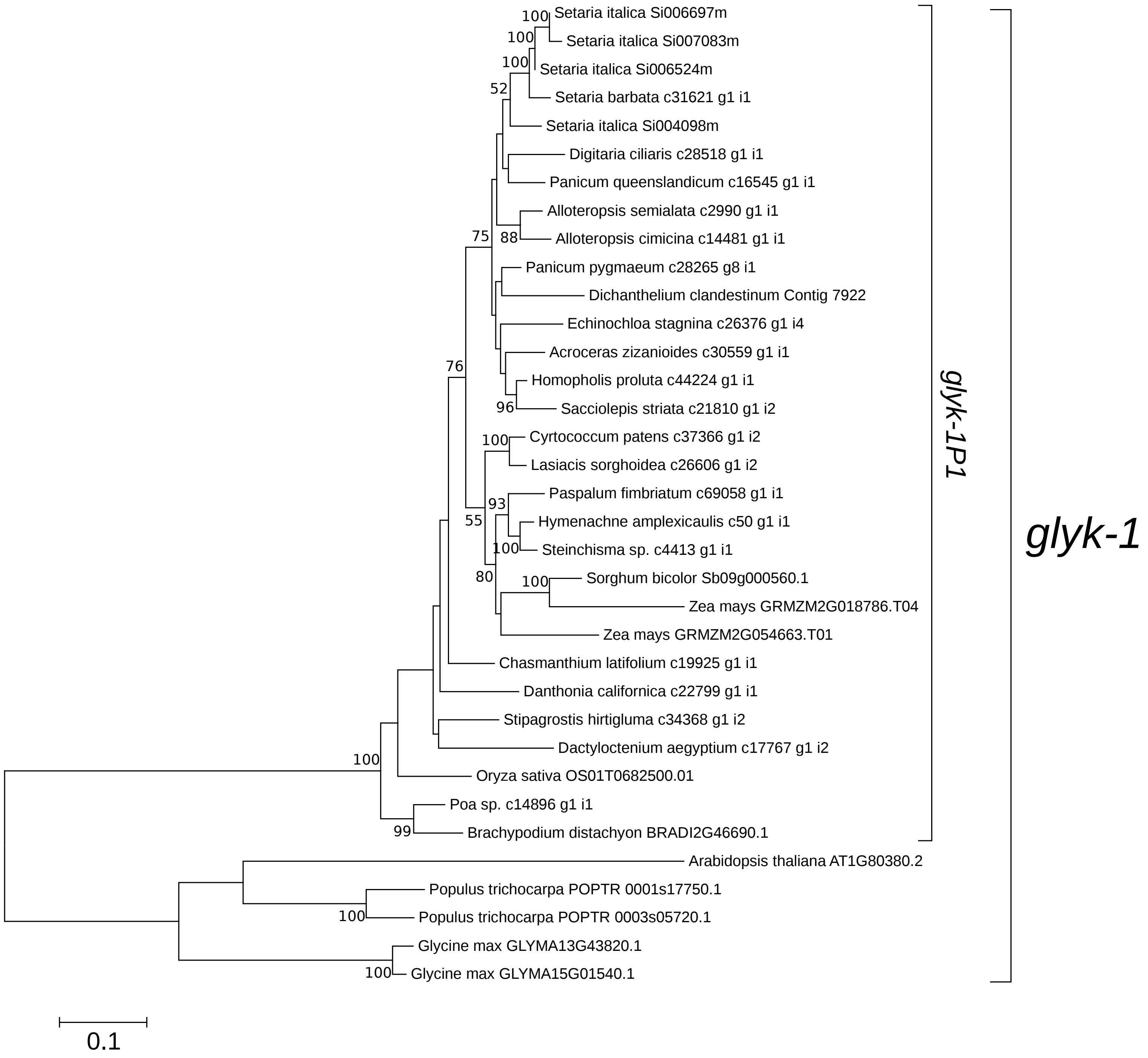

Glutathione reductase (GR) / Dihydrolipoyl dehydrogenase (Glycine Decarboxylase L protein) (LPD)

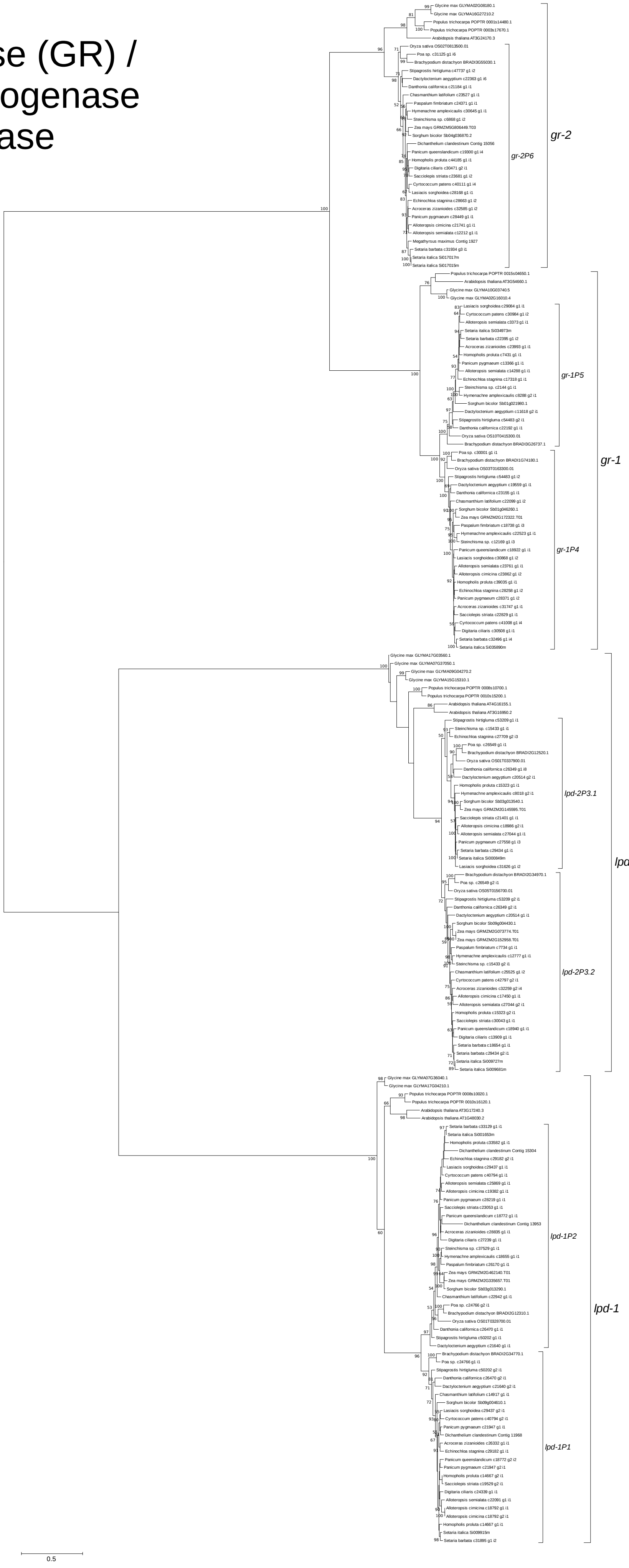

Glycerate dehydrogenase (glyoxylate/hydroxypyruvate reductase) (HPR)

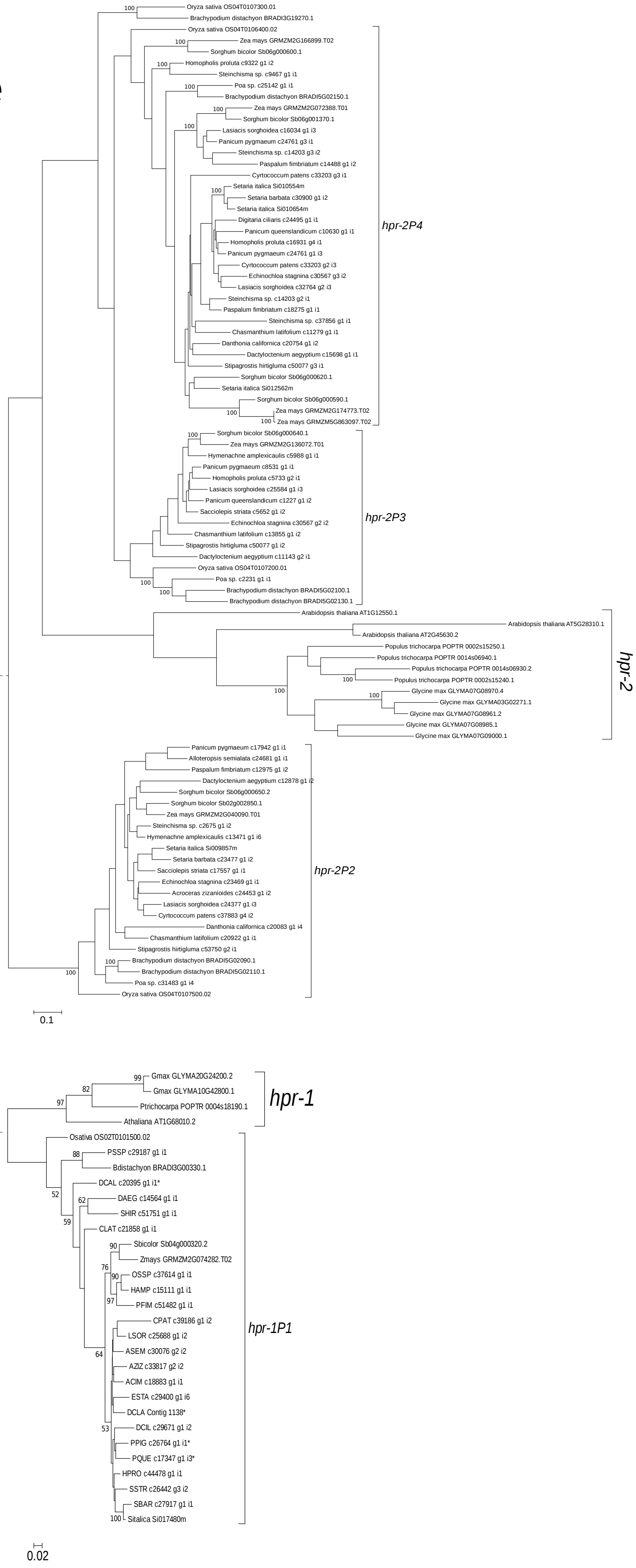

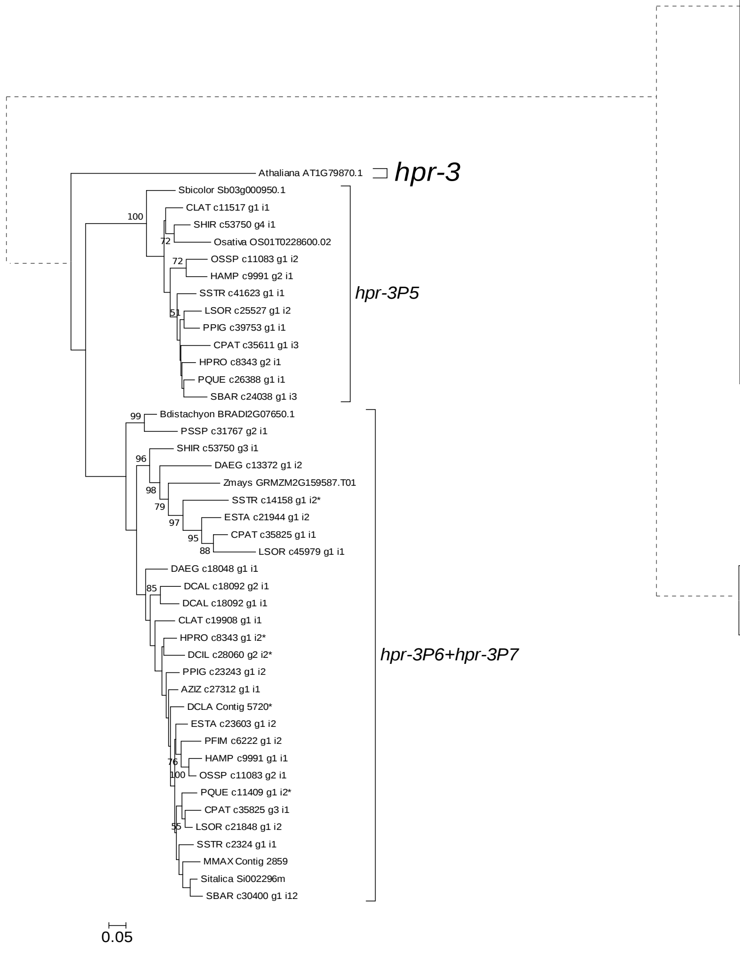

Phosphoglycolate phosphatase, chloroplastic (PGLP)

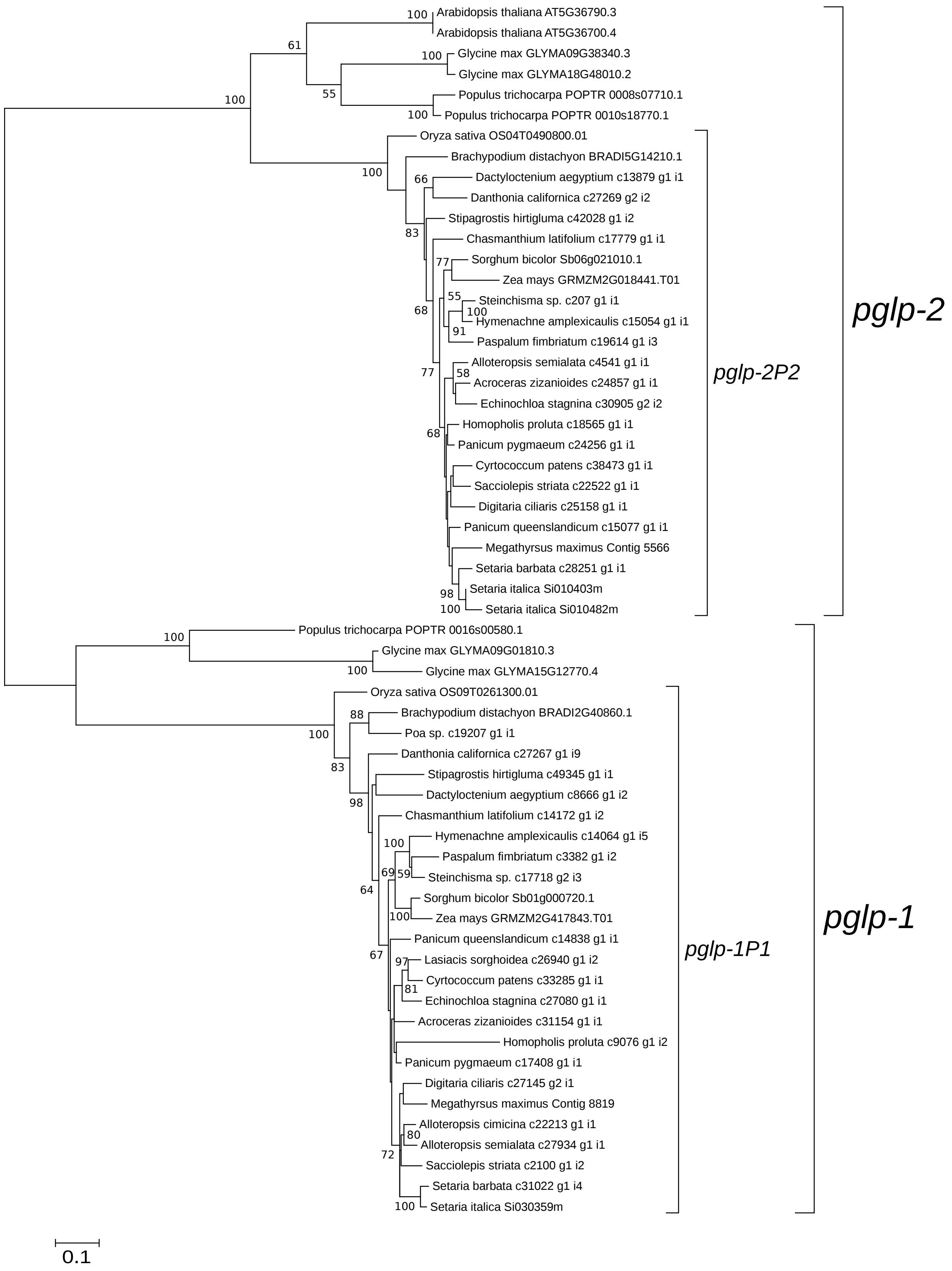

Serine hydroxymethyltransferase (SHM)

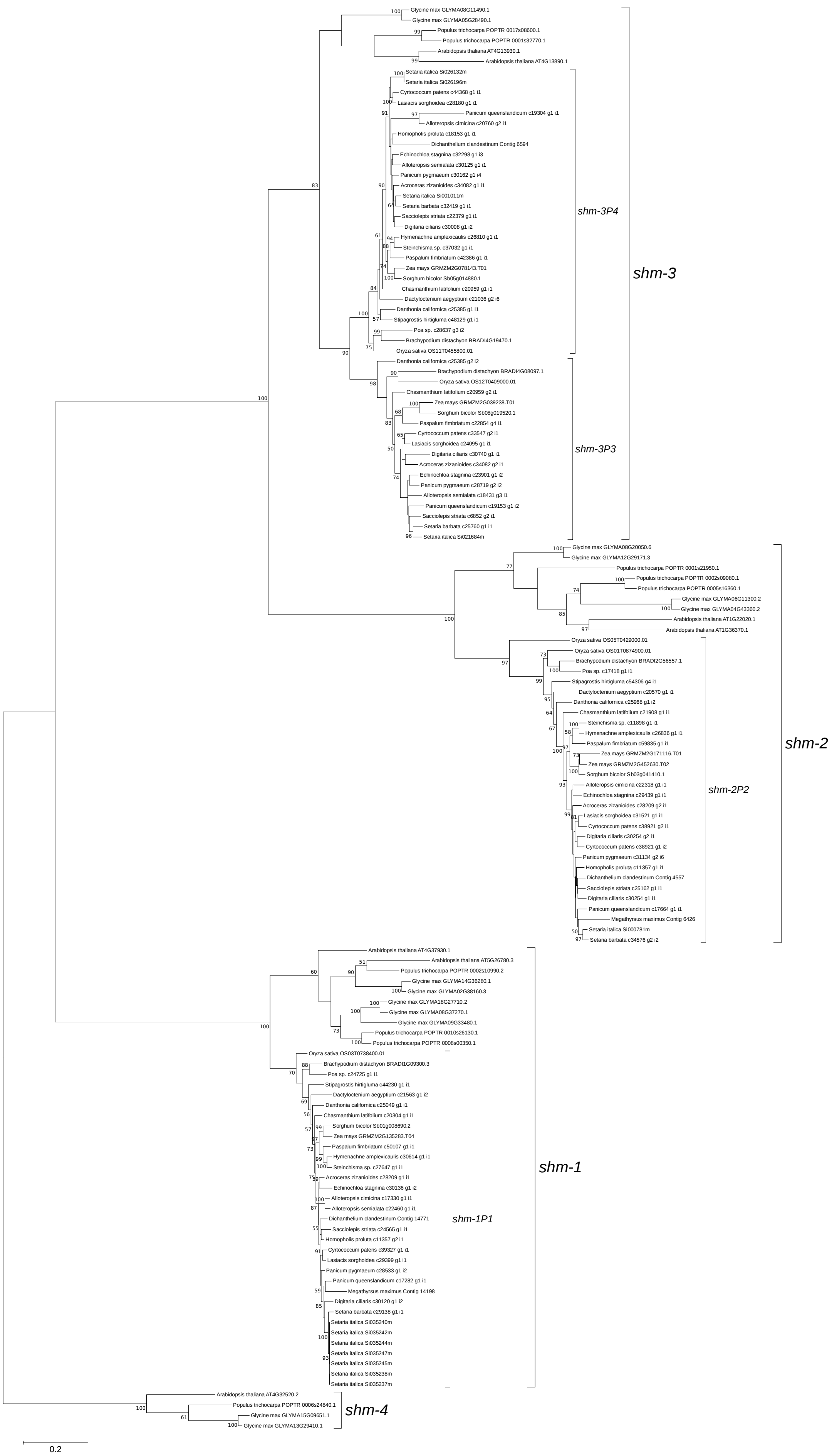

Table S1. **Sequencing statistics.**

Species	Abbre-viation	Tissue	PE Reads	Clean PE reads	Average reads	Contigs	Unigenes	N50 bp	Bowtie2 % CDS C4 genes
Acroceras zizanioides	AZIZ	Leaf	2202076	2035818	13048901	79597	53812	1514	2.66
	C_3	Leaf	3981203	3561989					2.1
		Root	5390799	5006883					1.98
		Root	2585123	2444211					1.19
Alloteropsis semialata	ASEM	Leaf	12422286	6546514	15218811	66427	47024	1591	7.4
	C_4	Leaf	1692525	1573457					4.26
		Root	3950750	3699333					1.53
		Root	3728547	3399507					1.11
Chasmanthium latifolium	CLAT	Leaf	10009259	8804513	23339126	64166	43050	1561	2.36
	C_3	Leaf	7933318	7136445					2.52
		Root	6440084	3148705					0.92
		Root	7522351	4249463					1.02
Cyrtococcum patens	CPAT	Leaf	8981967	6133295	33907966	120411	80962	1585	2.02
	C_3	Leaf	14553525	13258384					2.15
		Root	3367586	3162657					0.91
		Root	12455029	11353630					0.72
Dactyloctenium aegyptium	DAEG	Leaf	3562531	3255627	13266598	66023	44349	1661	2.58
	C_4	Leaf	8152613	7052648					3.46
		Root	3157583	2958323					0.88
Danthonia californica	DCAL	Leaf	3630245	3339890	15056375	76773	49480	1459	2.28
	C_3	Leaf	4639833	3945144					0.35
		Root	3342941	3082087					1.23
		Root	5574056	4689254					1.25
Digitaria ciliaris	DCIL	Leaf	4235388	3939771	15502945	55233	37500	1227	5.56
	C_4	Leaf	10424187	9195628					5.22
		Root	3949391	2015687					0.9
		Root	5814392	351859					0.31
Echinochloa stagnina	ESTA	Leaf	3191447	2903644	21321519	92233	66162	1458	6.99
	C_4	Leaf	11843314	10218776					6.91
		Root	1523231	1404008					1.12
		Root	7633573	6795091					0.83
Homopholis proluta	HPRO	Leaf	5729677	5134661	35973954	71089	52987	1888	3.11
	C_3	Leaf	11960661	10845052					2.18
		Root	10504783	9515317					0.91
		Root	11439945	10478924					0.89
Hymenachne amplexicaulis	HAMP	Leaf	3005381	2786048	12297065	53157	41303	1750	2.38
	C_3	Leaf	3007338	2269640					1.92
		Root	4241006	3931459					0.95
		Root	3569544	3309918					1.18
Lasiacis sorghoidea	LSOR	Leaf	2430756	2002336	27324623	103318	65626	1729	1.35
	C_3	Leaf	10391118	8957761					1.47
		Root	8533238	7936961					1.12
		Root	9610224	8427565					1.12

Panicum pygmaeum	PPYG	Leaf	4093890	3793221	17115317	72117	49086	1455	3.14
	C_3	Leaf	8925603	8087404					1.93
		Root	4106624	3482683					0.63
		Root	2026469	1752009					0.75
Panicum queenslandicum	PQUE	Leaf	3149789	2744427	11295659	54568	37682	1533	3.27
	C_4	Leaf	7280068	6630484					3.46
		Root	2590629	753302					1.27
		Root	3219178	1167446					0.11
Paspalum fimbriatum	PFIM	Leaf	3395354	3089206	21948286	96581	79239	1557	3.78
	C_4	Leaf	6499178	5782979					3.73
		Root	4306982	3919145					1.65
		Root	9895376	9156956					0.54
Poa sp.	PSSP	Leaf	4211710	3854186	23749106	83414	58393	1611	3.95
	C_3	Leaf	6384791	5587353					3.06
		Root	5060069	4639668					1.17
		Root	12291082	9667899					0.89
Sacciolepis striata	SSTR	Leaf	3148919	2867772	13833935	65538	43362	1639	2.41
	C_3	Leaf	7664481	6497248					2.59
		Root	4066769	3780174					0.98
		Root	868241	688741					0.31
Setaria barbata	SBAR	Leaf	3466394	3135585	16114099	81043	49155	1460	4.93
	C_4	Leaf	13286332	11936681					2.92
		Root	2508897	1041833					1.35
Steinchisma sp.	OSSP	Leaf	1536814	1417371	21852346	68171	50211	1906	3.29
	C_3	Leaf	8263727	7181006					2.26
		Root	5965474	5508124					0.87
		Root	8826368	7745845					0.86
Stipagrostis hirtigluma	SHIR	Leaf	14766660	14241012	72201207	141897	102685	1475	4.19
	C_4	Leaf	11712943	11296169					6.06
		Root	16975997	16292662					1.36
		Root	31560980	30371364					1.09

Table S3: Gene expression levels and co-option in two groups of eudicots.

Gene	Family	*Tarenaya spinosa*[1,2]	*Gynandropsis gynandra*[1,3]	Cleomaceae coopted	*Flaveria pringlei*[1,2]	*Flaveria robusta*[1,2]	*Flaveria australasica*[1,3]	*Flaveria* coopted
ak-1	AK	4.2	72.1	0	32.0	92.1	350.1	0
ak-2	AK	124.3	1055.0	1	104.6	96.5	168.7	0
alaat-1	ALA-AT	135.5	3862.9	1	55.3	125.1	966.7	1
aspat-1	ASP-AT	8.4	2895.2	1	90.4	69.3	24.2	0
aspat-2	ASP-AT	57.6	42.9	0	67.7	88.0	537.1	1
aspat-3	ASP-AT	6.7	33.6	0	128.4	128.1	180.0	0
dic-1	DIC	505.3	286.3	0	341.1	218.8	70.5	0
dic-2	DIC	0.0	459.7	0	25.5	23.1	8.0	0
dit-1	DIT	189.1	187.1	0	123.1	166.3	260.1	0
dit-2	DIT	60.7	5.2	0	50.7	58.1	96.7	0
nadmdh-1	NAD-MDH	82.9	39.2	0	62.7	42.3	39.7	0
nadmdh-2	NAD-MDH	170.1	217.8	0	173.5	180.2	136.1	0
nadmdh-3	NAD-MDH	1069.0	425.4	0	894.7	953.2	275.4	0
nadme-1	NAD-ME	39.3	813.0	1	53.3	71.0	68.7	0
nadme-2	NAD-ME	22.6	602.3	1	79.7	67.3	56.8	0
nadpmdh-1	NADP-MDH	169.4	262.8	0	229.9	372.4	2397.9	1
nadpmdh-2	NADP-MDH	2.9	4.1	0	16.4	43.6	2.5	0
nadpmdh-3	NADP-MDH	1137.5	446.1	0	437.2	578.4	643.0	0
nadpme-1E1	NADP-ME	24.2	91.4	0	133.0	368.3	2690.9	1
nadpme-1E2	NADP-ME	109.7	50.4	0	1.5	19.5	0.1	0
nadpme-1E3	NADP-ME	0.0	0.0	0	196.2	158.9	513.8	0
nhd-1	NHD	39.1	621.6	1	33.1	86.6	440.4	0
pck-1	PCK	8.9	60.6	0	9.3	25.3	19.0	0
pepck-1	PEPC-K	26.9	175.1	0	6.7	55.5	183.4	0
ppa-1	Ppa	717.5	2294.6	1	407.1	343.6	591.3	1
ppa-2	Ppa	82.7	229.1	0	46.8	52.3	176.2	0
ppa-3	Ppa	64.0	74.8	0	36.9	36.5	99.0	0
ppa-4	Ppa	310.4	137.4	0	7.5	10.9	13.9	0
ppc-1E1	PEPC	36.3	2841.0	1	0.0	54.0	1.6	0
ppc-1E2	PEPC	74.0	28.1	0	157.4	755.2	8302.4	1
ppc-2	PEPC	2.9	0.0	0	9.6	9.2	2.4	0
ppdk-1	PPDK	4.4	1022.9	1	160.8	471.8	5741.5	1
ppdkrp-1	PPDK-RP	44.9	111.7	0	20.7	83.0	321.8	0
ppt-1E1	TPT	16.3	0.0	0	176.8	243.1	186.9	0
ppt-1E2	TPT	58.9	1187.0	1	76.7	119.7	600.5	1
sbas-1	SBAS	39.0	3388.2	1	171.3	389.4	3420.0	1
sbas-2	SBAS	35.1	10.7	0	23.8	26.4	21.7	0
sbas-3	SBAS	10.5	3.0	0	17.6	39.2	4.5	0
sbas-4	SBAS	0.0	0.0	0	13.2	6.9	10.5	0
tpt-1E1	TPT	NA	NA	NA	0.2	68.3	1.4	0
tpt-1E2	TPT	654.1	2846.9	0	1103.6	755.1	895.1	1

[1] Expression levels are indicated for each gene, in rpkm values; [2] C_3 species; [3] C_4 species;

Table S4. Statistical models of co-option events for two groups of eudicots.

**C_4 lineage**	**Cleomaceae**		***Flaveria***	
**Factors**	**la[2]**	**family[3]**	**la[2]**	**family[3]**
**p-value**	0.19	0.39	0.01	0.84
**df[1]**	1,13	9,13	1,16	8,16
**F-stat**	1.87	1.17	8.17	0.49

[1] df = degrees of freedom. For each variable, the degrees of freedom for the residuals are given after the comma; [2] la = leaf abundance in close C_3 relatives; [3] gene family identity.

Table S5. Results of codon models comparisons.

gene	M1a	A	A1	p (M1a vs A1)[1]	p (A vs A1)[1]
ak-1P1	-4987.15	-4986.06	-4986.06	0.336	1.000
alaat-1P5	-9087.17	-9086.62	-9085.65	0.217	0.162
aspat-2P3	-7876.86	-7876.86	-7876.86	1.000	1.000
aspat-3P4	-7014.93	-7007.31	-7007.31	0.000*	1.000
bca-2P3	-6077.19	-6065.60	-6065.32	0.000*	0.457
dit-2P3	-6734.09	-6733.82	-6733.82	0.766	1.000
nadpmdh-1P1	-6195.23	-6194.90	-6194.90	0.717	0.923
nadpmdh-3P4	-5526.46	-5526.46	-5526.46	1.000	1.000
nadpme-1P4	-11513.59	-11461.08	-11460.45	0.000*	0.261
nhd-1P1	-8313.08	-8312.22	-8308.42	0.010	0.006
pck-1P1	-10293.72	-10184.91	-10184.91	0.000*	1.000
pepck-1P1	-2080.55	-2079.40	-2078.91	0.193	0.321
ppa-1P2.1	-4347.99	-4347.97	-4347.97	0.978	1.000
ppc-1P3	-18420.90	-18319.22	-18312.37	0.000*	0.000*
ppc-1P6	-11066.51	-11034.80	-11034.80	0.000*	1.000
ppdk-1P2	-18091.43	-18045.71	-18032.27	0.000*	0.000*
ppt-1P5	-5425.61	-5425.46	-5425.40	0.809	0.727
sbas-1P1	-5718.98	-5697.67	-5677.22	0.000*	0.000*
tpt-1P1	-5468.10	-5445.68	-5433.08	0.000*	0.000*

[1] p-values based on likelihood ratio tests. Significant comparisons after correction for multiple testing are indicated with an asterisk.
